# A contrast-adaptive method for simultaneous whole-brain and lesion segmentation in multiple sclerosis

**DOI:** 10.1016/j.neuroimage.2020.117471

**Published:** 2020-10-22

**Authors:** Stefano Cerri, Oula Puonti, Dominik S. Meier, Jens Wuerfel, Mark Mühlau, Hartwig R. Siebner, Koen Van Leemput

**Affiliations:** a Department of Health Technology, Technical University of Denmark, Denmark; b Danish Research Centre for Magnetic Resonance, Copenhagen University Hospital Hvidovre, Denmark; c Medical Image Analysis Center (MIAC AG) and Department of Biomedical Engineering, University Basel, Switzerland; d Department of Neurology and TUM-Neuroimaging Center, School of Medicine, Technical University of Munich, Germany; e Department of Neurology, Copenhagen University Hospital Bispebjerg, Denmark; f Institute for Clinical Medicine, Faculty of Medical and Health Sciences, University of Copenhagen, Denmark; g Athinoula A. Martinos Center for Biomedical Imaging, Massachusetts General Hospital, Harvard Medical School, USA

**Keywords:** Lesion segmentation, Multiple sclerosis, Whole-brain segmentation, Generative model

## Abstract

Here we present a method for the simultaneous segmentation of white matter lesions and normal-appearing neuroanatomical structures from multi-contrast brain MRI scans of multiple sclerosis patients. The method integrates a novel model for white matter lesions into a previously validated generative model for whole-brain segmentation. By using separate models for the shape of anatomical structures and their appearance in MRI, the algorithm can adapt to data acquired with different scanners and imaging protocols without retraining. We validate the method using four disparate datasets, showing robust performance in white matter lesion segmentation while simultaneously segmenting dozens of other brain structures. We further demonstrate that the contrast-adaptive method can also be safely applied to MRI scans of healthy controls, and replicate previously documented atrophy patterns in deep gray matter structures in MS. The algorithm is publicly available as part of the open-source neuroimaging package FreeSurfer.

## 1. Introduction

Multiple sclerosis (MS) is the most frequent chronic inflammatory autoimmune disorder of the central nervous system, causing progressive damage and disability. The disease affects nearly half a million Americans and 2.5 million individuals world-wide (Goldenberg, 2012; Rosati, 2001), generating more than $10 billion in annual healthcare spending in the United States alone (Adelman et al., 2013).

The ability to diagnose MS and track its progression has been greatly enhanced by magnetic resonance imaging (MRI), which can detect characteristic brain lesions in white and gray matter (Bakshi et al., 2008; Blystad et al., 2015; García-Lorenzo et al., 2013; Lövblad et al., 2010). Lesions visualized by MRI are up to an order of magnitude more sensitive in detecting disease activity compared to clinical assessment (Filippi et al., 2006). The prevalence and dynamics of white matter lesions are thus used clinically to diagnose MS (Thompson et al., 2018), define disease stages and to determine the efficacy of a therapeutic regimen (Sormani, 2013). MRI is also an unparalleled tool for characterizing brain atrophy, which occurs at a faster rate in patients with MS compared to healthy controls (Azevedo et al., 2018; Barkhof et al., 2009) and, especially in deep gray matter structures and the cerebral cortex, has been shown to correlate with measures of disability (Geurts et al., 2012).

Although manual labeling remains the most accurate way^1^ of delineating white matter lesions in MS (Commowick et al., 2018), this approach is very cumbersome and in itself prone to considerable intra- and inter-rater disagreement (Zijdenbos et al., 1998). Furthermore, manually labeling various normal-appearing brain structures to assess atrophy is simply too time consuming to be practically feasible. Therefore, there is a clear need for automated tools that can reliably and efficiently characterize the morphometry of white matter lesions, various neuroanatomical structures, and their changes over time directly from in vivo MRI. Such tools are of great potential value for diagnosing disease, tracking progression, and evaluating treatment. They can also help in obtaining a better understanding of underlying disease mechanisms, and to facilitate more efficient testing in clinical trials. Ultimately, automated software tools may help clinicians to prospectively identify which patients are at highest risk of future disability accrual, leading to better counseling of patients and better overall clinical outcomes.

Despite decades of methodological development (cf. García-Lorenzo et al., 2013 or Danelakis et al., 2018), currently available computational tools for analyzing MRI scans of MS patients remain limited in a number of important ways:

- Poor generalizability: Existing tools are often developed and tested on very specific imaging protocols, and may not be able to work on data that is acquired differently. Especially with the strong surge of supervised learning in recent years, where the relationship between image appearance and segmentation labels in training scans is directly and statically encoded, the segmentation performance of many state-of-the-art algorithms will degrade substantially when applied to data from different scanners and acquisition protocols (García-Lorenzo et al., 2013; Valverde et al., 2019), severely limiting their usefulness in practice.
- Dearth of available software: Despite the very large number of proposed methods, most algorithms are only developed and tested inhouse, and very few tools are made publicly available (Griffanti et al., 2016; Schmidt et al., 2012; Shiee et al., 2010; Valverde et al., 2017). In order to secure that computational methods will make a real practical impact, they must be accompanied by software implementations that work robustly across a wide array of image acquisitions; that are made publicly available; and that are open-sourced, rigorously tested and comprehensively documented.
- Limitations in assessing atrophy: There is a lack of dedicated tools for characterizing brain atrophy patterns in MS: many existing methods characterize only aggregate measures such as global brain or gray matter volume (Smeets et al., 2016; Smith et al., 2002) rather than individual brain structures, or require that lesions are pre-segmented so that their MRI intensities can be replaced with placeholder values to avoid biased atrophy measures (Azevedo et al., 2018; Battaglini et al., 2012; Ceccarelli et al., 2012; Chard et al., 2010; Gelineau-Morel et al., 2012; Sdika and Pelletier, 2009) (so-called lesion filling).

**Fig. 1.**
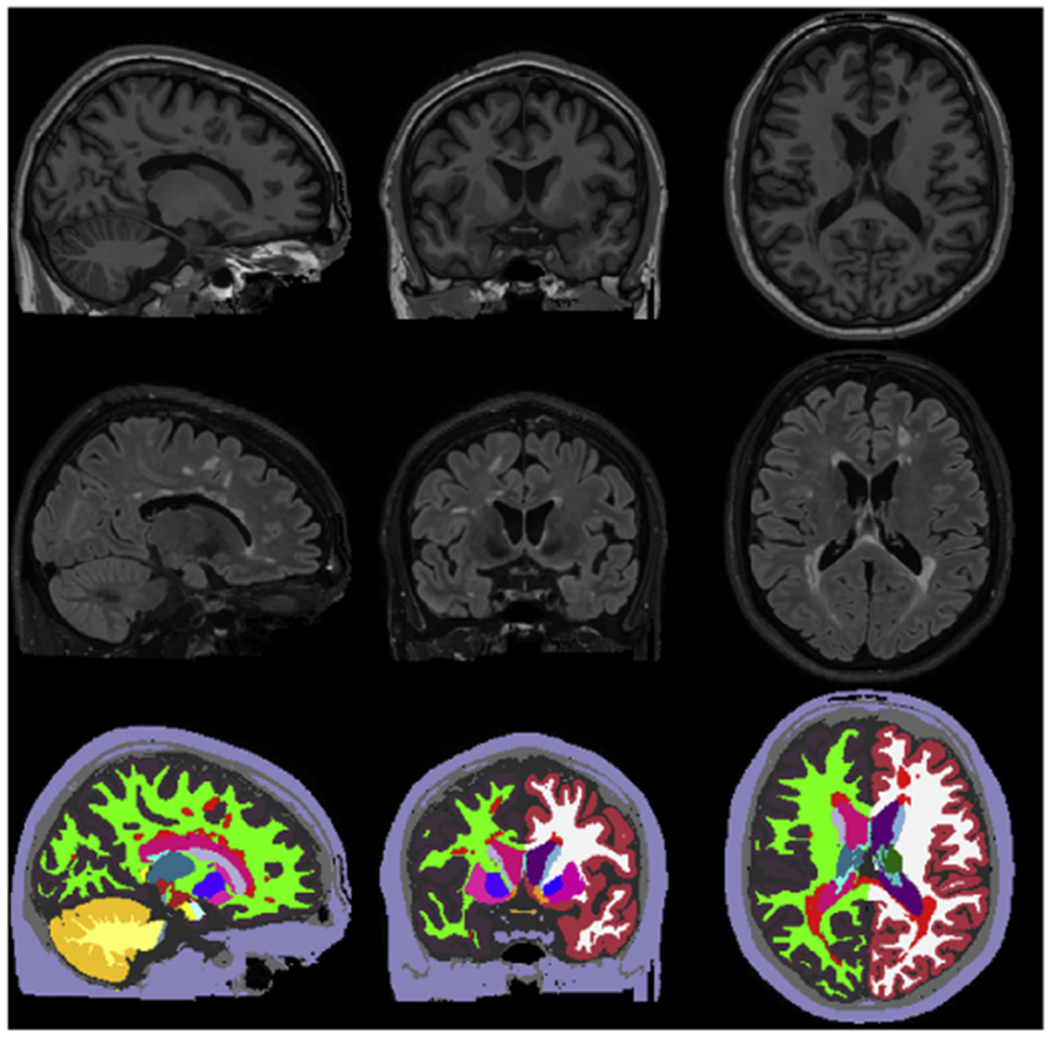
Segmentation of white matter lesions and 41 different brain structures from the proposed method on T1w-FLAIR input. From left to right: sagittal, coronal, axial view. From top to bottom: T1w, FLAIR, automatic segmentation.

In order to address these limitations, we describe a new open-source software tool for simultaneously segmenting white matter lesions and 41 neuroanatomical structures from MRI scans of MS patients. An example segmentation produced by this tool is shown in Fig. 1. By performing lesion segmentation in the full context of whole-brain modeling, the method obviates the need to segment lesions and assess atrophy in two separate processing phases, as currently required in lesion filling approaches. The method works robustly across a wide range of imaging hardware and protocols by completely decoupling computational models of anatomy from models of the imaging process, thereby sidestepping the intrinsic generalization difficulties of supervised methods such as convolutional neural networks. Our software implementation is freely available as part of the FreeSurfer neuroimaging analysis package (Fischl, 2012).

To the best of our knowledge, only two other methods have been developed for joint whole-brain and white matter lesion segmentation in MS. Shiee et al. (2010) model lesions as an extra tissue class in an unsupervised whole-brain segmentation method (Bazin and Pham, 2008), removing false positive detections of lesions using a combination of topological constraints and hand-crafted rules implementing various intensity- and distance-based heuristics. However, the method segments only a small set of neuroanatomical structures (10), and validation of this aspect was limited to a simulated MRI scan of a single subject. McKinley et al. (2019) use a cascade of two convolutional neural networks, with the first one skull-stripping individual image modalities and the second one generating the actual segmentation. However, the whole-brain segmentation performance of this method was only evaluated on a few structures (7). Furthermore, as a supervised method its applicability on data that differs substantially from its training data will necessarily be limited.

A preliminary version of this work was presented in Puonti and Van Leemput (2016). Compared to this earlier work, the current article employs more advanced models for the shape and appearance of white matter lesions, and includes a more thorough validation of the segmentation performance of the proposed method, including an evaluation of the whole-brain segmentation component and comparisons with human inter-rater variability.

## 2. Contrast-adaptive whole-brain segmentation

We build upon a method for whole-brain segmentation called Sequence Adaptive Multimodal SEGmentation (SAMSEG) that we previously developed (Puonti et al., 2016), and that we propose to extend with the capability to handle white matter lesions. SAMSEG robustly segments 41 structures from head MRI scans without any form of preprocessing or prior assumptions on the scanning platform or the number and type of pulse sequences used. Since we build heavily on this method for the remainder of the paper, we briefly outline its main characteristics here.

**Fig. 2.**
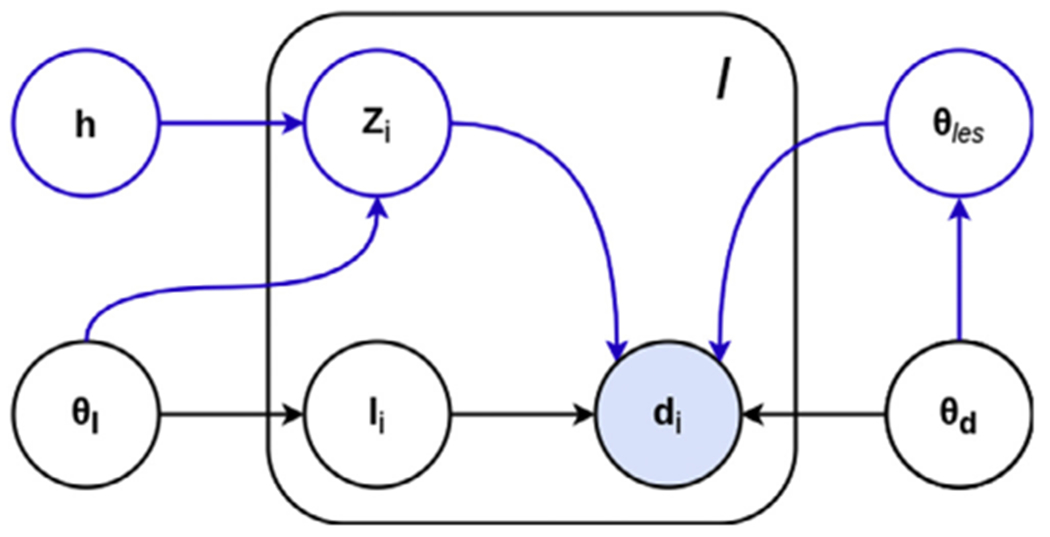
Graphical model of the proposed method. In black the existing contrast-adaptive whole-brain segmentation method SAMSEG (without lesion modeling), in blue the proposed additional components to also model white matter lesions. Shading indicates observed variables. The plate indicates *I* repetitions of the included variables, where *I* is the number of voxels.

SAMSEG is based on a generative approach, in which a forward probabilistic model is inverted to obtain automated segmentations. Let **D** = (**d**_1_ , …, **d***_I_*) denote a matrix collecting the intensities in a multi-contrast brain MR scan with *I* voxels, where the vector $d_{i}={\left(d_{i}^{1},\ldots ,d_{N}^{i}\right)}^{T}$ contains the intensities in voxel *i* for each of the available *N* contrasts. Furthermore, let **l** = (*l*_1_ , …, *l_I_*)^*T*^ be the corresponding labels, where *l_i_* ∈ {1 , … *K*} denotes one of the *K* possible segmentation labels assigned to voxel *i*. SAMSEG estimates a segmentation **l** from MRI data **D** by using a generative model, illustrated in black in Fig. 2. According to this model, **l** is sampled from a segmentation prior *p*(**l**|***θ*_l_**), after which **D** is obtained by sampling from a likelihood function *p*(**D|l, *θ*_d_**), where ***θ*_l_** and ***θ*_d_** are model parameters with priors *p*(***θ*_l_**) and *p*(***θ*_d_**). Segmentation then consists of inferring the unknown **l** from the observed **D** under this model. In the following, we summarize the segmentation prior and the likelihood used in SAMSEG, as well as the way the resulting model is used to obtain automated segmentations.

### 2.1. Segmentation prior

To model the spatial configuration of various neuroanatomical structures, we use a deformable probabilistic atlas as detailed in Puonti et al. (2016). In short, the atlas is based on a tetrahedral mesh, where the parameters ***θ_l_*** are the spatial positions of the mesh’s vertices, and *p*(***θ_l_***) is a topology-preserving deformation prior that prevents the mesh from tearing or folding (Ashburner et al., 2000). The model assumes conditional independence of the labels between voxels for a given deformation:

$$p\left(l|\theta_{1}\right)=\prod_{i=1}^{I}p\left(l_{i}|\theta_{l}\right),$$

and computes the probability of observing label *k* at voxel *i* as

(1) $$p\left(l_{i}=k|\theta_{l}\right)=\sum_{j=1}^{J}\alpha_{j}^{k}\psi_{j}^{i}\left(\theta_{l}\right),$$

where $a_{k}^{j}$ are label probabilities defined at the *J* vertices of the mesh, and $\psi_{j}^{i}(\theta_{l})$ denotes a spatially compact, piecewise-linear interpolation basis function attached to the *j^th^* vertex and evaluated at the *i^th^* voxel (Van Leemput, 2009).

The topology of the mesh, the mode of the deformation prior *p*(***θ_l_***), and the label probabilities $\alpha_{k}^{j}$ can be learned automatically from a set of segmentations provided as training data (Van Leemput, 2009). This involves an iterative process that combines a mesh simplification operation with a group-wise nonrigid registration step to warp the atlas to each of the training subjects, and an Expectation Maximization (EM) algorithm (Dempster et al., 1977) to estimate the label probabilities $\alpha_{k}^{j}$ in the mesh vertices. The result is a sparse mesh that encodes high-dimensional atlas deformations through a compact set of vertex displacements. As described in Puonti et al. (2016), the atlas used in SAMSEG was derived from manual whole-brain segmentations of 20 subjects, representing a mix of healthy individuals and subjects with questionable or probable Alzheimer’s disease.

### 2.2. Likelihood function

For the likelihood function we use a Gaussian model for each of the *K* different structures. We assume that the bias field artifact can be modelled as a multiplicative and spatially smooth effect (Wells et al., 1996). For computational reasons, we use log-transformed image intensities in **D**, and model the bias field as a linear combination of spatially smooth basis functions that is added to the local voxel intensities (Van Leemput et al., 1999). Letting ***θ*_d_** collect all bias field parameters and Gaussian means and variances, the likelihood is defined as

$$p(D|l,\theta_{d})=\prod_{i=1}^{I}p(d_{i}|l_{i},\theta_{d}),$$

$$p(d_{i}|l_{i}=k,\theta_{d})=N(d_{i}|\mu_{k}+C\phi_{i},\Sigma_{k}),$$

$$\begin{array}{l} C=\left(\begin{array}{c} c_{1}^{T}\\ \vdots \\ c_{N}^{T} \end{array}\right),\;c_{n}=\left(\begin{array}{c} c_{n,1}\\ \vdots \\ c_{n,P} \end{array}\right),\;\phi_{i}=\left(\begin{array}{c} \phi_{1}^{i}\\ \vdots \\ \phi_{P}^{i} \end{array}\right), \end{array}$$

where *P* denotes the number of bias field basis functions, $\phi_{p}^{i}$ is the basis function *p* evaluated at voxel *i*, and **c***_n_* holds the bias field coefficients for MRI contrast *n*. We use a flat prior for the parameters of the likelihood: *p*(***θ*_d_**) ∝ 1.

### 2.3. Segmentation

For a given MRI scan **D**, segmentation proceeds by computing a point estimate of the unknown model parameters ***θ*** = {***θ_d_, θ_l_***}:

$$\hat{\theta }=\arg\underset{\theta }{\;\max}\;p(\theta |D),$$

which effectively fits the model to the data. Details of this procedure are given in Appendix A. Once $\hat{\theta }$ is found, the corresponding maximum a posteriori (MAP) segmentation

$$\hat{l}=\arg\underset{I}{\;\max}\;p(l|D,\hat{\theta })$$

is obtained by assigning each voxel to the label with the highest probability, i.e., ${\hat{l}}_{i}=\arg\;\max_{k}\;{\hat{w}}_{i,k}$, where $0\leq {\hat{w}}_{i,k}\leq 1$ are probabilistic label assignments

(2) $$w_{i,k}=\frac{N(d_{i}|\mu_{k}+C_{\phi_{i}},\Sigma_{k})p(l_{i}=k|\theta_{l})}{\Sigma_{k\prime =1}^{K}N(d_{i}|\mu_{k\prime }+C\phi_{i},\Sigma_{k\prime })p(l_{i}=k\prime |\theta_{l})}$$

evaluated at the estimated parameters $\hat{\theta }$. It is worth emphasizing that, since the class means and variances {***μ**_k_*, **Σ**_*k*_} are estimated from each target scan individually, the model automatically adapts to each scan’s specific intensity characteristics – a property that we demonstrated experimentally on several data sets acquired with different imaging protocols, scanners and field strengths in Puonti et al. (2016).

Our implementation of this method, written in Python with the exception of C++ parts for the computationally demanding optimization of the atlas mesh deformation, is available as part of the open-source package FreeSurfer^2^. It segments MRI brain scans without any form of preprocessing such as skull stripping or bias field correction, taking around 10 minutes to process one subject on a state-of-the-art computer (measured on a machine with an Intel 12-core i7-8700K processor). As explained in Puonti et al. (2016), in our implementation we make use of the fact that many neuroanatomical structures share the same intensity characteristics in MRI to reduce the number of free parameters in the model (e.g., all white matter structures share the same Gaussian mean ***μ**_k_* and variance **Σ**_*k*_, as do most gray matter structures). Furthermore, for some structures (e.g., non-brain tissue) we use Gaussian mixture models instead of a single Gaussian. In addition to using full covariance matrices **Σ**_*k*_, our implementation also supports diagonal covariances, which is currently selected as the default behavior.

## 3. Modeling lesions

In order to make SAMSEG capable of additionally segmenting white matter lesions, we augment its generative model by introducing a binary lesion map **z** = (*z*_1_ , … , *z_I_*)^*T*^, where *z_i_* ∈ {0, 1} indicates the presence of a lesion in voxel *i*. The augmented model is depicted in Fig. 2, where the blue parts indicate the additional components compared to the original SAMSEG method. The complete model consists of a joint (i.e., over both **l** and **z** simultaneously) segmentation prior *p*(**l, z|h, *θ*_l_**), where **h** is a new latent variable that helps constrain the *shape* of lesions, as well as a joint likelihood *p*(**D|l, z, *θ*_d_**, ***θ**_les_*), where ***θ**_les_* are new parameters that govern their *appearance*. In the following, we summarize the segmentation prior and the likelihood used in the augmented model, as well as the way the resulting model is used to obtain automated segmentations.

### 3.1. Segmentation prior

We use a joint segmentation prior of the form

$$p\left(l,z|h,\theta_{l}\right)=p\left(z|h,\theta_{l}\right)p\left(l|\theta_{l}\right),$$

where *p*(**l**|***θ*_l_**) is the deformable atlas model defined in Section 2.1, and

$$p\left(z|h,\theta_{l}\right)=\prod_{i=1}^{I}p\left(z_{i}|h,\theta_{l}\right)$$

is a factorized model where the probability that a voxel is part of a lesion is given by:

$$p\left(z_{i}=1|{|h,\theta }_{l}\right)=f_{i}\left(h\right)\rho_{i}\left(\theta_{l}\right).$$

Here 0 ≤ *f_i_*(**h**) ≤ 1 aims to enforce *shape* constraints on lesions, whereas 0 ≤ *ρ_i_*(**θ_l_**) ≤ 1 takes into account a voxel’s spatial *location* within its neuroanatomical context. Below we provide more details on both these components of the model.

#### 3.1.1. Modeling lesion shapes

In order to model lesion shapes, we use a variational autoencoder (Kingma and Welling, 2013; Rezende et al., 2014) according to which lesion segmentation maps **z** are generated in a two-step process: An unobserved, low-dimensional code **h** is first sampled from a spherical Gaussian distribution p(h)=N(h|0,I), and subsequently “decoded” into **z** by sampling from a factorized Bernoulli model:

$$p_{\omega }\left(z|h\right)=\prod_{i=1}^{I}f_{i}{\left(h\right)}^{z_{i}}{\left(1-f_{i}\left(h\right)\right)}^{\left(1-z_{i}\right)}.$$

Here *f_i_*(**h**) are the outputs of a “decoder” convolutional neural network (CNN) with filter weights ***ω***, which parameterize the model.

Given a training data set in the form of *N* binary segmentation maps $D={\left\{z^{\left(n\right)}\right\}}_{n=1}^{N},$, suitable network parameters ***ω*** can in principle be estimated by maximizing the log-probability assigned to the data by the model :

$$\log\;p_{\omega }\left(D\right)=\sum_{z\in D}\log\;p_{\omega }\left(z\right),\;\mathrm{where}\;p_{\omega }\left(z\right)=\int_{h}p_{\omega }\left(z_{n}|h\right)p\left(h\right)\text{d}h.$$

However, because the integral over the latent codes makes this intractable, we use amortized variational inference in the form of stochastic gradient variational Bayes (Kingma and Welling, 2013; Rezende et al., 2014). In particular, we introduce an approximate posterior

$$q_{\upsilon }\left(h|z\right)=N\left(h|\mu_{\upsilon }\left(z\right),\;\mathrm{diag}\left(\sigma_{\upsilon }^{2}\left(z\right)\right)\right),$$

where the functions ***μ**_υ_*(**z**) and ***ρ**_υ_*(**z**) are implemented as an “encoder” CNN parameterized by ***υ***. The variational parameters ***υ*** are then learned jointly with the model parameters ***ω*** by maximizing a variational lower bound $\sum_{z\in D}L_{\omega ,\upsilon }\left(z\right)\leq \log\;p_{\omega }\left(D\right)$ using stochastic gradient descent, where

(3) $$L_{\omega ,\upsilon }\left(z\right)=-D_{KL}\left(q_{\upsilon }\left(h|z\right)||p\left(h\right)\right)+E_{q_{\upsilon }\left(h|z\right)}\left[\log\;p_{\omega }\left(z|h\right)\right].$$

The first term is the Kullback–Leibler divergence between the approximate posterior and the prior, which can be evaluated analytically. The expectation in the last term is approximated using Monte Carlo sampling, using a change of variables (known as the “reparameterization trick”) to reduce the variance in the computation of the gradient with respect to ***υ*** (Kingma and Welling, 2013; Rezende et al., 2014).

**Fig. 3.**
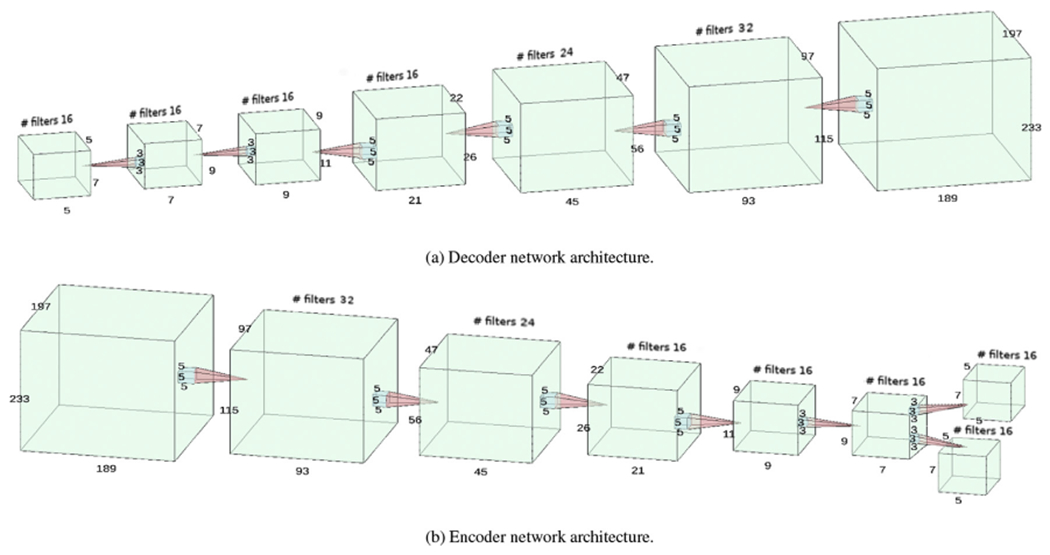
Lesion shape model architecture consisting of two symmetrical convolutional neural networks: (a) decoder network and (b) encoder network. The decoder network generates lesion segmentations from a low-dimensional code. Its architecture has ReLU activation functions (*f*(*x*) = *max*(0, *x*)) and batch normalization (Ioffe and Szegedy, 2015) between each deconvolution layer, with the last layer having a sigmoid activation function, ensuring 0 ≤ *f_i_* (**h**) ≤ 1. The encoder network encodes lesion segmentations into a latent code. The main differences compared to the decoder network are the use of convolutional layers instead of deconvolutional layers and, to encode the mean and variance parameters, the last layer has been split in two, with no activation function for the mean and a softplus activation function (*f*(*x*) = ln(1 + *e^x^*)) for the variance.

Our training data set D was derived from manual lesion segmentations in 212 MS subjects, obtained from the University Hospital of Basel, Switzerland. The segmentations were all affinely registered and resampled to a 1 mm isotropic grid of size 197×233×189. In order to reduce the risk of overfitting to the training data, we augmented each segmentation in the training data set by applying a rotation of 10 degrees around each axis, obtaining a total of 1484 segmentations. The architecture for our encoder and decoder networks is detailed in Fig. 3. We trained the model for 1000 epochs with mini-batch size of 10 using Adam optimizer (Kingma and Ba, 2014) with a learning rate of 1e-4. We approximated the expectation in the variational lower bound of Eq. (3) by using a single Monte Carlo sample in each step.

#### 3.1.2. Modeling the spatial location of lesions

In order to encode the spatially varying frequency of occurrence of lesions across the brain, we model the probability of finding a lesion in voxel *i*, based on its location alone, as

$$\rho_{i}\left(\theta_{l}\right)=\sum_{j=1}^{J}\beta_{j}\psi_{j}^{i}\left(\theta_{l}\right),$$

where lesion probabilities 0 ≤ *β_j_* ≤ 1 defined in the vertices of the SAM-SEG atlas mesh are interpolated at the voxel location. This effectively defines a lesion probability map that deforms in conjunction with the SAMSEG atlas to match the neuroanatomy in each image being segmented, allowing the model to impose contextual constraints on where lesions are expected to be found.

We estimated the parameters *β_j_* by running SAMSEG on MRI scans (T1-weighted (T1w) and FLAIR) of 54 MS subjects in whom lesions had been manually annotated (data from the University Hospital of Basel, Switzerland), and recording the estimated atlas deformations. The parameters *β_j_* were then computed from the manual lesion segmentations by applying the same technique we used to estimate the $\alpha_{j}^{k}$ parameters in the SAMSEG atlas training phase (cf. Section 2.1).

### 3.2. Likelihood function

For the likelihood, which links joint segmentations {**l**, **z**} to intensities **D**, we use the same model as SAMSEG in voxels that do not contain lesion (*z_i_* = 0), but draw intensities in lesions (*z_i_* = 1) from a separate Gaussian with parameters ***θ**_les_* = {***μ**_les_*, **Σ***_les_*}:

$$p\left(D|l,z,\theta_{d},\theta_{les}\right)=\prod_{i=1}^{I}p\left(d_{i}|l_{i},z_{i},\theta_{d},\theta_{les}\right),$$

where

$$p\left(d_{i}|l_{i}=k,z_{i},\theta_{d},\theta_{les}\right)=\{\begin{array}{cc} N\left(d_{i}|\mu_{les}+C\phi_{i},\Sigma_{les}\right) & \mathrm{if}\;z_{i}=1,\\ N\left(d_{i}|\mu_{k}+C\phi_{i},\Sigma_{k}\right) & \mathrm{otherwise}. \end{array}$$

In order to constrain the values that the lesion intensity parameters ***θ**_les_* can take, we make them conditional on the remaining intensity parameters using a normal-inverse-Wishart distribution :

(4) $$p\left(\theta_{les}|\theta_{d}\right)=N\left(\mu_{les}|\mu_{WM},\nu^{-1}\Sigma_{les}\right)\mathrm{IW}\left(\Sigma_{les}|\kappa \nu \Sigma_{WM},\nu -N-2\right).$$

Here the subscript “WM” denotes the white matter Gaussian and *κ* > 1 and *ν* ≥ 0 are hyperparameters in the model.

This choice of model is motivated by the fact that the normal-inverse-Wishart distribution is a conjugate prior for the parameters of a Gaussian distribution: Eq. (4) can be interpreted as providing *ν* “pseudo-voxels” with empirical mean ***μ**_W M_* and variance *κ***Σ***_W M_* in scenarios where the lesion intensity parameters ***μ**_les_* and **Σ***_les_* need to be estimated from data. In the absence of any such pseudo-voxels (*ν* = 0), Eq. (4) reduces to a flat prior on *θ_les_* and lesions are modeled as a completely independent class. Although such models have been used in the literature (Guttmann et al., 1999; Kikinis et al., 1999; Shiee et al., 2010; Sudre et al., 2015) their robustness may suffer when applied to subjects with no or very few lesions, such as controls or patients with early disease, since there is essentially no data to estimate the lesion intensity parameters from. In the other extreme case, the number of pseudo-voxels can be set to such a high value (*ν* → ∞) that the intensity parameters of the lesions are fully determined by those of WM. This effectively replaces the Gaussian intensity model for WM in SAMSEG by a distribution with longer tails, in the form of a mixture of two Gaussians with identical means (***μ**_les_* ≡ ***μ**_W M_*) but variances that differ by a constant factor (**Σ**_*les*_ ≡ *κ***Σ***_W M_* vs. **Σ***_W M_*). In this scenario, MS lesions are detected as model outliers in a method using robust model parameter estimation (Huber, 1981), another technique that has also frequently been used in the literature (Aït-Ali et al., 2005; Bricq et al., 2008; García-Lorenzo et al., 2011; Liu et al., 2009; Prastawa and Gerig, 2008; Rousseau et al., 2008; Van Leemput et al., 2001).

Based on pilot experiments on a variety of datasets (distinct from the ones used in the results section), we found that good results are obtained by using an intermediate value of *ν* = 500 pseudo-voxels for 1 mm^3^ isotropic scans, together with a scaling factor *κ* = 50. In order to adapt to different image resolutions, *ν* is scaled inversely proportionally with the voxel size in our implementation. We will visually demonstrate the role of these hyperparameters in constraining the lesion intensity parameters in Section 5.1.

### 3.3. Segmentation

As in the original SAMSEG method, segmentation proceeds by first obtaining point estimates $\hat{\theta }$ that fit the model to the data, and then inferring the corresponding segmentation posterior:

$$p\left(l,z|D,\hat{\theta }\right),$$

which is now jointly over **l** and **z** simultaneously. Unlike in SAMSEG, however, both steps are made intractable by the presence of the new variables ***θ**_les_* and **h** in the model. In order to side-step this difficulty, we obtain $\hat{\theta }$ through a joint optimization over both ***θ*** and ***θ**_les_*:

$$\left\{\hat{\theta },{\hat{\theta }}_{les}\right\}=\arg\;\underset{\left\{\theta ,\theta_{les}\right\}}{\max}p\left(\theta ,\theta_{les}|D\right)$$

in a simplified model in which the constraints on lesion *shape* have been removed, by clamping all decoder network outputs *f_i_*(**h**) to value 1. This simplification is defensible since the aim here is merely to find appropriate model parameters, rather than highly accurate lesion segmentations. By doing so, the latent code **h** is effectively removed from the model and the optimization simplifies into the one used in the original SAM-SEG method, with only minor modifications due to the prior *p*(***θ***_*les*_|***θ*_d_**). Details are provided in Appendix B.

Once parameter estimates $\hat{\theta }$ are available, we compute segmentations using the factorization

$$p\left(l,z|D,\hat{\theta }\right)=p\left(z|D,\hat{\theta }\right)p\left(l|,z,D,\hat{\theta }\right),$$

first estimating **z** from $p\left(z|D,\hat{\theta }\right)$ (Step 1 below), and then plugging this into $p\left(l|,z,D,\hat{\theta }\right)$ to estimate **l** (Step 2):

Step 1: Evaluating $p\left(z|D,\hat{\theta }\right)$ involves marginalizing over both **h** and ***θ**_les_*, which we approximate by drawing *S* Monte Carlo samples ${\left\{h^{\left(s\right)},\theta_{les}^{\left(s\right)}\right\}}_{s=1}^{S}$ from $p\left(h,\theta_{les}|D,\hat{\theta }\right)$:

$$p\left(z|D,\hat{\theta }\right)=\int_{h,\theta_{les}}p\left(z|D,\hat{\theta },h,\theta_{les}\right)p\left(h,\theta_{les}|D,\hat{\theta }\right)dh,\theta_{les}\;≃\frac{1}{S}\sum_{s=1}^{S}p\left(z|D,\hat{\theta },h^{\left(s\right)},\theta_{les}^{\left(s\right)}\right).$$

This allows us to estimate the probability of lesion occurrence in each voxel, which we then compare with a user-specified threshold value *γ*

$$p\left(z_{i}=1|d_{i},\hat{\theta }\right)≷\gamma$$

to obtain the final lesion segmentation ${\hat{z}}_{i}$. Details on how we approximate $p\left(z_{i}=1|d_{i},\hat{\theta }\right)$ using Monte Carlo sampling are provided in Appendix C.

Step 2: Voxels that are not assigned to lesion (${\hat{z}}_{i}=0$) in the previous step are finally assigned to the neuroanatomical structure with the highest probability $p(l_{i}=k|z_{i}=0,d_{i},\hat{\theta }),$, which simply involves computing ${\hat{l}}_{i}={\arg\;\max}_{k}\;{\hat{w}}_{i,k}$ with ${\hat{w}}_{i,k}$ defined in Eq. (2).

In agreement with other work (Aït-Ali et al., 2005; García-Lorenzo et al., 2011; Jain et al., 2015; Prastawa and Gerig, 2008; Shiee et al., 2010; Van Leemput et al., 2001), we have found that using known prior information regarding the expected intensity profile of MS lesions in various MRI contrasts can help reduce the number of false positive detections. Therefore, we prevent some voxels from being assigned to lesion (i.e., forcing ${\hat{z}}_{i}=0$) based on their intensities in relation to the estimated intensity parameters ${\left\{{\hat{\mu }}_{k},{\hat{\Sigma }}_{k}\right\}}_{k=1}^{K}:$: In our current implementation only voxels with an intensity higher than the mean of the gray matter Gaussian in FLAIR and/or T2 (if these modalities are present) are considered candidate lesions.

Since estimating $p\left(z_{i}=1|d_{i},\hat{\theta }\right)$ involves repeatedly invoking the decoder and encoder networks of the lesion shape model, as detailed in Appendix C, we implemented the proposed method as an add-on to SAMSEG in Python using the Tensorflow library (Abadi et al., 2015). Estimating $\hat{\theta }$ has the same computational complexity as running SAM-SEG (i.e., taking approximately 10 minutes on a state-of-the-art machine with an Intel 12-core i7-8700K CPU), while the Monte Carlo sampling takes an additional 5 minutes on a GeForce GTX 1060 graphics card, bringing the total computation time to around 15 minutes per subject.

## 4. Evaluation datasets and benchmark methods

In this section, we describe four datasets that we will use for the experiments in this paper, including two taken from public challenges. We also outline two relevant methods for MS lesion segmentation that the proposed method is compared to in detail, as well as the metrics and measures used in our experiments.

### 4.1. Datasets

In order to test the proposed method and demonstrate its contrast-adaptiveness, we conducted experiments on four datasets acquired with different scanner platforms, field strengths, acquisition protocols and image resolution:

- **MSSeg**: This dataset is the publicly available training set of the MS lesion segmentation challenge that was held in conjunction with the MICCAI 2016 conference (Commowick et al., 2018). It consists of 15 MS cases from three different scanners, all acquired using a harmonized imaging protocol (Cotton et al., 2015). For each patient a 3D T1w sequence, a contrast-enhanced (T1c) sequence, an axial dual PD-T2-weighted (T2w) sequence and a 3D fluid attenuation inversion recovery (FLAIR) sequence were acquired. Each subject’s lesions were delineated by seven different raters on the FLAIR scan and, if necessary, corrected using the T2w scan. These delineated images were then fused to create a consensus lesion segmentation for each subject. Both raw images and pre-processed images (pre-processing steps: denoising, rigid registration, brain extraction and bias field correction – see Commowick et al. (2018) for details) were made available by the challenge organizers. In our experiments we used the pre-processed data, which required only minor modifications in our software to remove non-brain tissues from the model. We note that the original challenge also included a separate set of 38 test subjects, but at the time of writing this data is no longer available.
- **Trio**: This dataset consists of 40 MS cases acquired on a Siemens Trio 3T scanner at the Danish Research Center of Magnetic Resonance (DRCMR). For each patient, a 3D T1w sequence, a T2w sequence and a FLAIR sequence were acquired. Ground truth lesion segmentations were automatically delineated on the FLAIR images using Jim software^3^, and then checked and, if necessary, corrected by and expert rater at DRCMR using the T2w and MPRAGE images.
- **Achieva**: This dataset consists of 50 MS cases and 25 healthy controls acquired on a Philips Achieva 3T scanner at DRCMR. After a visual inspection of the images, we decided to remove 2 healthy controls from the dataset as they present marked gray matter atrophy and white matter hyperintensities. For each patient, a 3D T1w sequence, a T2w sequence and a FLAIR sequence were acquired. Ground truth lesion segmentations were delineated using the same protocol as the one used for the Trio dataset.
- **ISBI**: This dataset is the publicly available test set of the MS lesion segmentation challenge that was held at the 2015 International Symposium on Biomedical Imaging (Carass et al., 2017). It consists of 14 longitudinal MS cases, with 4 to 6 time points each, separated by approximately one year. Images were acquired on a Philips 3T scanner. For each patient, a 3D T1w sequence, a T2w sequence, a PDw sequence and a FLAIR sequence were acquired. Images were first preprocessed (inhomogeneity correction, skull stripping, dura stripping, again inhomogeneity correction – see Carass et al. (2017) for details), and then registered to a 1 mm MNI template. Each subject’s lesions were delineated by two different raters on the FLAIR scan, and, if necessary, corrected using the other contrasts. As part of the challenge, a training dataset of 5 additional longitudinal MS cases is also available, with the same scanner, imaging protocols and delineation procedure as the test dataset.

A summary of the datasets, with scanner type, image modalities and voxel resolution details, can be found in Table 1. For each subject all the contrasts were co-registered and resampled to the FLAIR scan for MSSeg, and to the T1w scan for Trio, Achieva and ISBI. This is the only preprocessing step required by the proposed method.

### 4.2. Benchmark methods for lesion segmentation

In order to evaluate the lesion segmentation component of the proposed method in detail, we compared it to two publicly available and widely used algorithms for MS lesion segmentation:

- **LST-lga**^4^ (Schmidt et al., 2012): This lesion growth algorithm starts by segmenting a T1w image into three main tissue classes (CSF, GM and WM) using SPM12^5^, and combines the resulting segmentation with co-registered FLAIR intensities to calculate a lesion belief map. A pre-chosen initial threshold *κ* is then used to create an initial binary lesion map, which is subsequently grown along voxels that appear hyperintense in the FLAIR image. We set *κ* to its recommended default value of 0.3, which was also used in previous studies (Mühlau et al., 2013; Rissanen et al., 2014).
- **NicMsLesions**^6^ (Valverde et al., 2017, 2019): This deep learning method is based on a cascade of two 3D convolutional neural networks, where the first one reveals possible candidate lesion voxels, and the second one reduces the number of false positive outcomes. Both networks were trained by the authors of the method on T1w and FLAIR scans coming from a publicly available training dataset of the MS lesion segmentation challenge held in conjunction with the MICCAI 2008 conference (Styner et al., 2008) (20 cases) and the MSSeg dataset (15 cases). This method was one of the top performers on the test dataset of the MICCAI 2016 challenge (Commowick et al., 2018), and one of the few methods for which an implementation is publicly available.

We note that both these benchmark methods are specifically targeting T1w-FLAIR input, whereas the proposed method is not tuned to any particular combination of input modalities.

Although we only compared our method in detail to these two benchmarks, many more good methods for MS lesion segmentation exist. We refer the reader to the MSSeg paper (Commowick et al., 2018), the ISBI challenge paper (Carass et al., 2017) and the ISBI challenge website^7^ to compare the reported performance further with other ones.

### 4.3. Metrics and measures

In order to evaluate the influence of varying the input modalities on the segmentation performance of the proposed method, and to assess segmentation accuracy with respect to that of other methods and human raters, we used a combination of segmentation volume estimates, Pearson correlation coefficients between such estimates and reference values, and Dice scores. Volumes were computed by counting the number of voxels assigned to a specific structure and converting into mm^3^ , whereas Dice coefficients were computed as

$${\mathrm{Dice}}_{X,Y}=\frac{2\cdot \left|X\cap Y\right|}{\left|X\right|+\left|Y\right|},$$

where *X* and *Y* denote segmentation masks, and | · | counts the number of voxels in a mask.

The proposed method and both benchmark algorithms produce a probabilistic lesion map that needs to be thresholded to obtain a final lesion segmentation. This requires an appropriate threshold value to be set for this purpose (variable *γ* in the proposed method). In order to ensure an objective comparison between the methods, we used a leave-one-out cross-validation strategy in which the threshold for each test image was set to the value that maximizes the average Dice overlap with manual segmentations in all the other images of the same dataset. For the reported performance of the methods on the ISBI dataset, the thresholds were tuned on the 5 training subjects that are part of the challenge instead.

## 5. Results

In this section, we first illustrate the effect of the various components of our model. We then evaluate how the proposed model adapts to different input modalities and acquisition platforms. Subsequently we compare the lesion segmentation performance of our model against that of the two benchmark methods, relate it to human inter-rater variability, and analyze its performance on the ISBI challenge data. Finally, we perform an indirect validation of the whole-brain segmentation component of the method.

Throughout the section we use boxplots to show some of the results. In these plots, the median is indicated by a horizontal line, plotted inside boxes that extend from the first to the third quartile values of the data. The range of the data is indicated by whiskers extending from the boxes, with outliers represented by circles.

### 5.1. Illustration of the method

**Fig. 4.**
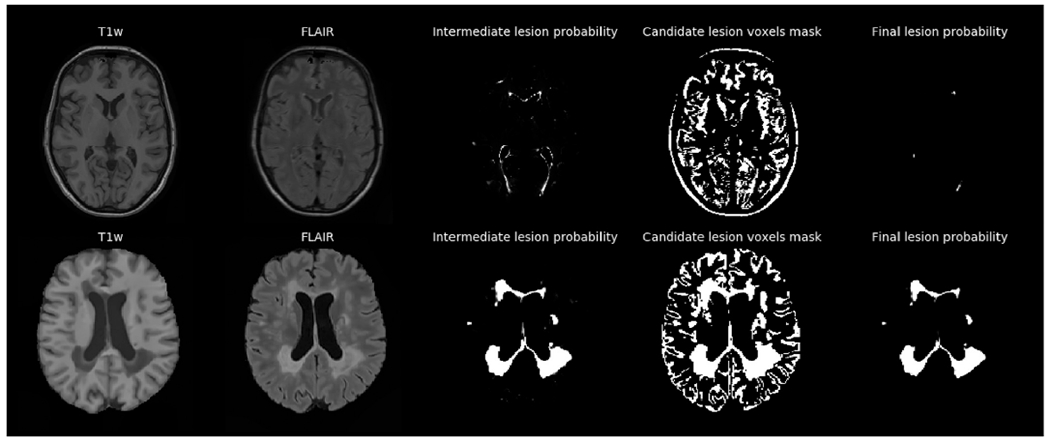
Illustration of how intensity constraints and the lesion shape model help reduce false positive lesion detections in the method. Top row: a subject with a low lesion load; Bottom row: a subject with a high lesion load. From left to right: T1w and FLAIR input; intermediate lesion probability obtained with the simplified model used to estimate $\hat{\theta }$; mask of candidate voxels based on intensity alone (intensity higher than the mean gray matter intensity in FLAIR); and final lesion probability estimate $p\left(z_{i}=1|d_{i},\hat{\theta }\right)$ produced by the method.

In order to illustrate the effect of the various components of the method, here we analyze its behaviour when segmenting T1w-FLAIR scans of two MS subjects – one with a low and one with a high lesion load. Fig. 4 shows, in addition to the input data and the final lesion probability estimate $p\left(z_{i}=1|d_{i},\hat{\theta }\right)$, also an intermediate lesion probability obtained with the simplified model used to estimate $\hat{\theta }$, i.e., before the FLAIR-based intensity constraints and the lesion shape constraints are applied. From these images we can see that the lesion shape model and the intensity constraints help remove false positive detections and enforce more realistic shapes of lesions, especially for the case with low lesion load.

**Fig. 5.**
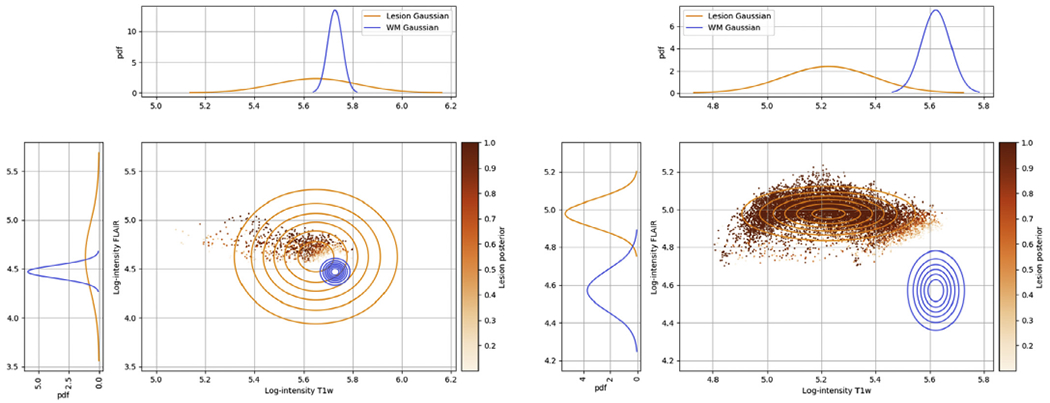
Illustration of the effect of the prior *p*(***θ**_les_*|***θ*_d_**) on the lesion intensity parameters, both in the case of a lesion load that is low (left, corresponding to the subject in the top row of Fig. 4) and high (right, corresponding to the subject in the bottom row of Fig. 4). The illustration is from the Monte Carlo sampling phase of the method: In each case, the value of the parameters of the lesion Gaussian is taken as the average over the Monte Carlo samples ${\left\{\theta_{les}^{\left(s\right)}\right\}}_{s=1}^{S},$ and the points represent the resulting lesion posterior estimate $p\left(z_{i}=1|d_{i},\hat{\theta }\right)$ in each voxel.

Fig. 5 analyzes the effect of the prior *p*(***θ**_les_*|***θ*_d_**) on the lesion intensity parameters ***θ**_les_* for the two subjects shown in Fig. 4. When the lesion load is high, the prior does not have a strong influence, leaving the lesion Gaussian “free” to fit the data. However, when the lesion load is low, the lesion Gaussian is constrained to retain a wide variance and a mean close to the mean of WM, effectively turning the model into an outlier detection method for WM lesions. This behavior is important in cases when few lesions are present in the images, ensuring the method works robustly even when only limited data is available to estimate the lesion Gaussian parameters.

In order to analyze the effect of the lesion shape prior, we compared the lesion segmentation performance of the proposed method with that obtained when the shape prior was intentionally removed from the model (i.e., all the decoder network outputs *f_i_*(**h**) clamped to value 1). For a fair comparison, the lesion threshold value *γ* was re-tuned to maximize performance for the method without shape prior, in the way described in Section 4.3. Table 2 summarizes the results across the MSSeg, Trio and Achieva datasets, for different ranges of lesion load. In addition to Dice scores, the table also reports results for precision and recall, defined as

$$precision=\frac{TP}{TP+FP}\;recall=\frac{TP}{TP+FN},$$

where *TP, FP* and *FN* count the true positive, false positive and false negative voxels compared to the manual segmentation. The results indicate that, although performance is unchanged for high lesion loads, for which segmentation is generally easier (Commowick et al., 2018), the lesion shape prior clearly improves segmentations in subjects with small and medium lesion loads.

**Fig. 6.**
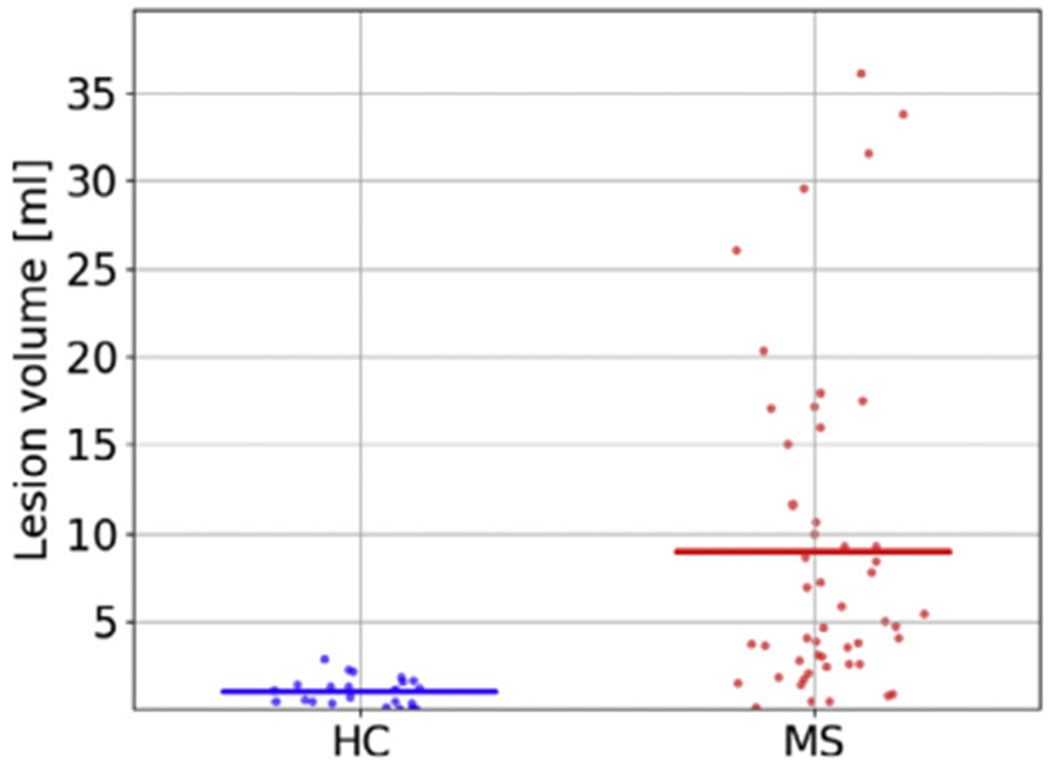
Difference between healthy controls (HC) and MS subjects in lesion volume, as detected by the proposed method on the Achieva dataset (23 HC subjects, 50 MS subjects, T1w-FLAIR input). Lines indicate means across subjects.

In order to demonstrate that the model also works robustly in control subjects (with no lesions at all), and can therefore be safely applied in studies comparing MS subjects with controls, we further segmented T1w-FLAIR scans of the Achieva dataset, and computed the total volume of the lesions in each subject. The results are shown in Fig. 6; the volumes were 8.95±9.18 ml for MS subjects vs. 0.98±0.77 ml for controls. Although the average lesion volume for controls was not exactly zero, a visual inspection revealed that this was due to some controls having WM hyperintensities that were segmented by the method as MS lesions, which we find acceptable.

### 5.2. Scanner and contrast adaptive segmentations

**Fig. 7.**
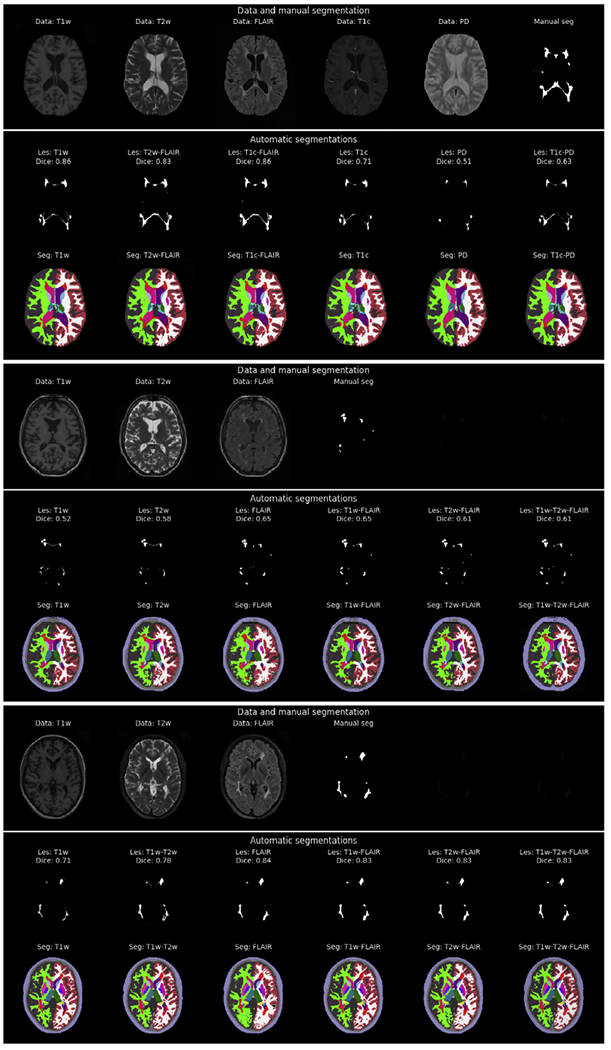
Contrast-adaptiveness of the proposed method to different combinations of input modalities. Segmentations are shown for one subject of the MSSeg (top row), the Trio (middle row) and the Achieva MS (bottom row) dataset. For each subject the top row shows slices of the data and the manual lesion annotation; the middle row shows the lesion probability map and Dice score computed by the proposed method for specific input combinations; and the bottom row shows the corresponding complete segmentations produced by the method. Enlarged figures for each subject are available in the Supplementary Material Figs. 1–3.

In order to demonstrate the ability of our method to adapt to different types and combinations of MRI sequences acquired with different scanners, we show the method’s segmentation results along with the manual segmentations for a representative subset of combinations for one subject in the MSSeg (consensus as manual segmentation), the Trio and the Achieva datasets in Fig. 7. It is not feasible to show all possible combinations. For instance, mixing the 5 contrasts in the MSSeg dataset alone already yields 31 possible multi-contrast combinations. Nonetheless, it is clear that the model is indeed able to adapt to the specific contrast properties of its input scans. A visual inspection of its whole-brain segmentation component seems to indicate that the method benefits from having access to the T1w contrast for best performance. This is especially clear when only the FLAIR contrast is provided, as this visually degrades the segmentation of the white-gray boundaries in the cortical regions due to the low contrast between white and gray matter in FLAIR.

**Fig. 8.**
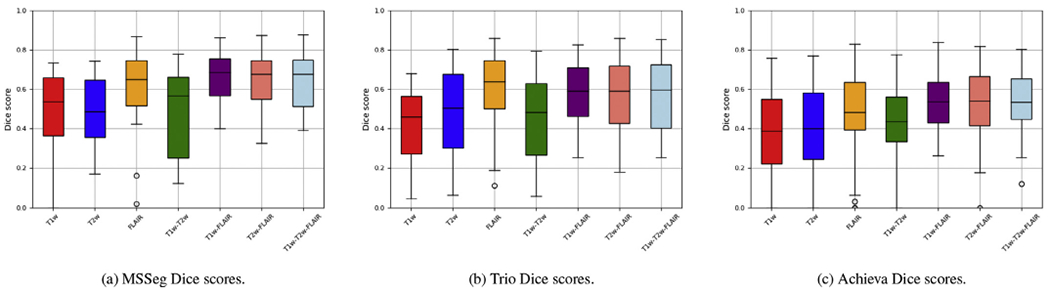
Lesion segmentation performance of the proposed method in terms of Dice overlap with manual raters on three different datasets when different input contrasts are used (T1w, T2w, FLAIR, T1w-T2w, T1w-FLAIR, T2w-FLAIR, T1w-T2w-FLAIR). From left to right: Dice scores on MSSeg, Trio and Achieva MS data.

When comparing the lesion probability maps produced by the method visually with the corresponding manual lesion segmentations, it seems that the method benefits from having access to the FLAIR contrast for the best lesion segmentation performance. This is confirmed by a quantitative analysis shown in Fig. 8, which plots the Dice overlap scores for each of the seven input combinations that all our three datasets have in common, namely T1w, T2w, FLAIR, T1w-T2w, T1w-FLAIR, T2w-FLAIR, and T1w-T2w-FLAIR. Although the inclusion of additional contrasts does not hurt lesion segmentation performance, across all three datasets the best results are obtained whenever the FLAIR contrast is included as input to the model. This finding is perhaps not surprising, given that the manual delineations were all primarily based on the FLAIR image.

Considering both the whole-brain and lesion segmentation performance together, we conclude that the combination T1w-FLAIR is well-suited for obtaining good results with the proposed method, although it will also accept other and/or additional contrasts beyond T1w and FLAIR.

### 5.3. Lesion segmentation

In order to compare the lesion segmentation performance of our model against that of the two benchmark methods, and relate it to human inter-rater variability, we here present a number of results based on the T1w-FLAIR input combination (which is the combination required by the benchmark methods). We also analyze the lesion segmentation performance of our method on the public ISBI challenge.

#### 5.3.1. Comparison with benchmark lesion segmentation methods

**Fig. 9.**
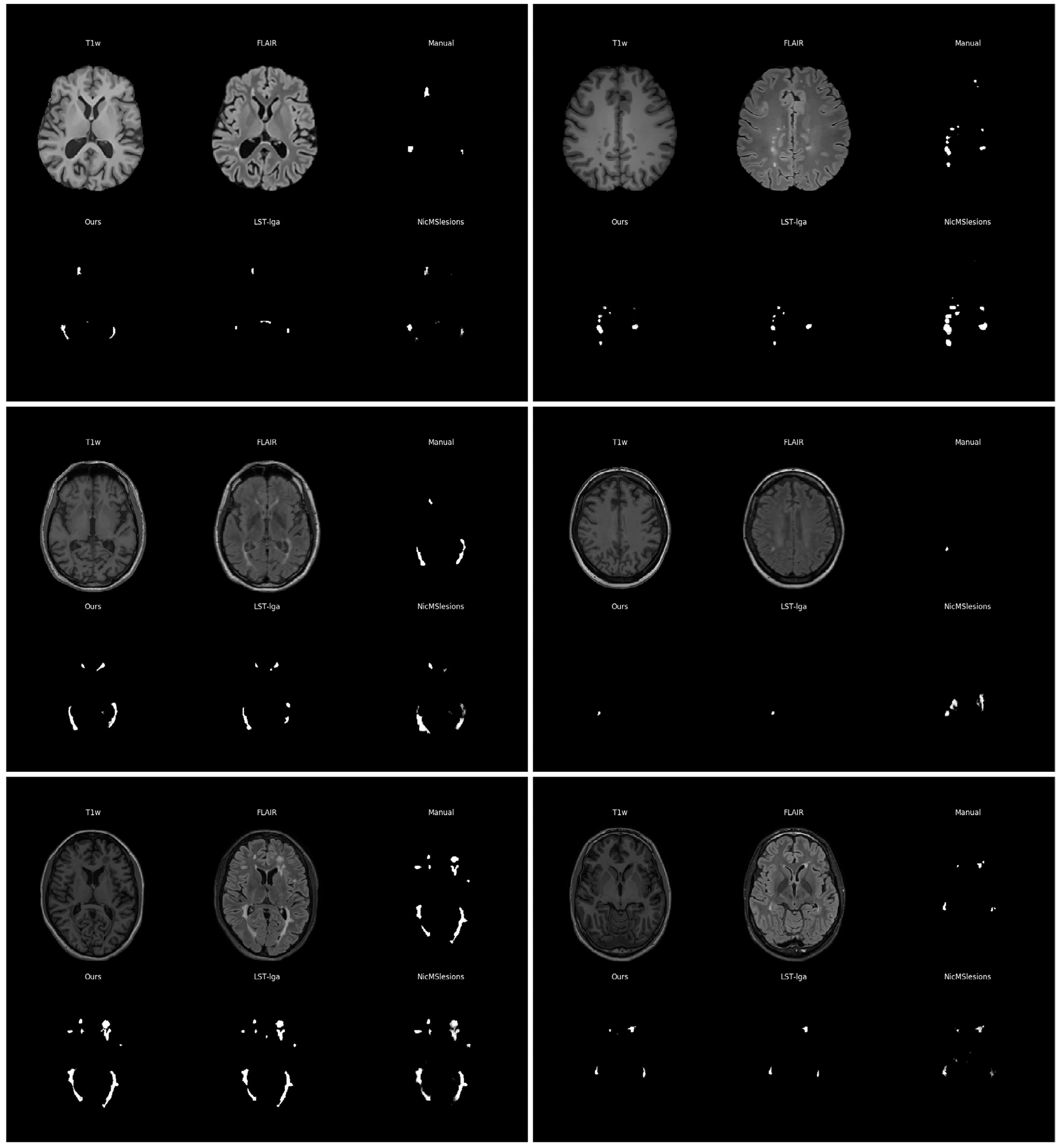
Visual comparison of lesion probability maps on three different datasets for the proposed method and two state-of-the-art lesion segmentation methods (LST-lga and NicMsLesions) on T1w-FLAIR input. (Top) Two subjects from the MSSeg dataset; (Middle) Two subjects from the Trio dataset; (Bottom) Two subjects from the Achieva dataset. For each subject the top row shows slices of the data and the manual annotation while the bottom row shows the lesion probability maps for our model, LST-lga and NicMsLesions.

**Fig. 10.**
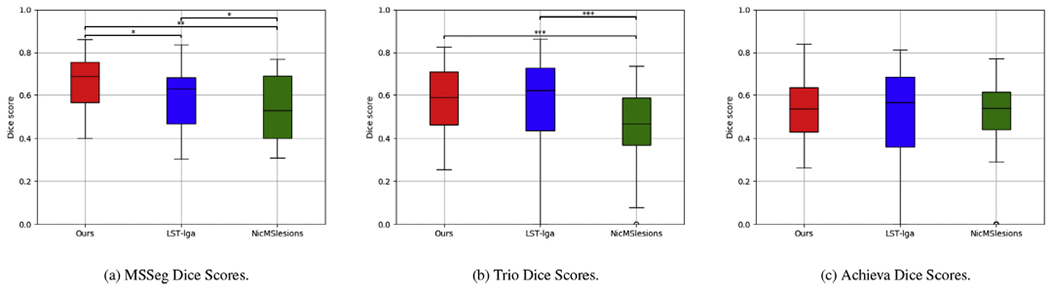
Lesion segmentation performance in terms of Dice overlap with manual raters for the proposed method and two benchmark methods (LST-lga and NicMsLesions) on T1w-FLAIR input. Statistically significant differences between two methods, computed with a two-tailed paired *t*-test, are indicated by asterisks (“***” for *p*-value < 0.001, “**” for *p*-value < 0.01 and “*” for *p*-value < 0.05). From left to right: results on the MSSeg, the Trio and the Achieva dataset.

Fig. 9 shows automatic segmentations of two randomly selected subjects from the MSSeg, the Trio and the Achieva datasets, both for our method and for the two benchmark methods LST-lga and NicMSLesions, along with the corresponding manual segmentations (consensus manual segmentations for MSSeg). Visually, all three methods perform similarly on the Achieva MS data, but some of the results for NicMSLesions appear to be inferior to those obtained with the other two methods on MSSeg and Trio data. This qualitative observation is confirmed by the quantitative analysis shown in Fig. 10, where the three methods’ Dice overlap scores are compared on each dataset: similar performances are obtained for all methods on the Achieva data, but NicMSLesions trails the other two methods on MSSeg and Trio data. Especially for MSSeg data this is a surprising result, since NicMSLesions was trained on this specific dataset, i.e., the subjects used for testing were part of the training data of this method, potentially biasing the results in favor of NicMSLesions. Based on Dice scores, the proposed method outperforms LST-lga on MSSeg data, although there are no statistically significant differences between the two methods on the other datasets.

#### 5.3.2. Results on the ISBI data

We also evaluated the performance of the proposed method on the ISBI challenge data, obtaining a mean Dice score of 0.58 when T1w-FLAIR input is used. This score is comparable to the ones we obtained on the other three datasets analyzed in this paper (cf. Fig. 10) – MSSeg: 0.65, Trio: 0.58 and Achieva: 0.54. A few example segmentation results on the ISBI data are available in the Supplementary Material, Fig. 4.

The ISBI challenge website^8^ ranks submissions according to an overall lesion segmentation performance score that takes into account Dice overlap, volume correlation, surface distance, and a few other metrics (see Carass et al., 2017 for details). A score of 100 indicates perfect correspondence, while 90 is meant to correspond to human inter-rater performance (Carass et al., 2017; Styner et al., 2008). We obtained a score of 87.87, which places us around half-way in the ranking of the original challenge (Carass et al., 2017), although we note that the website currently lists methods with a much higher score.

**Fig. 11.**
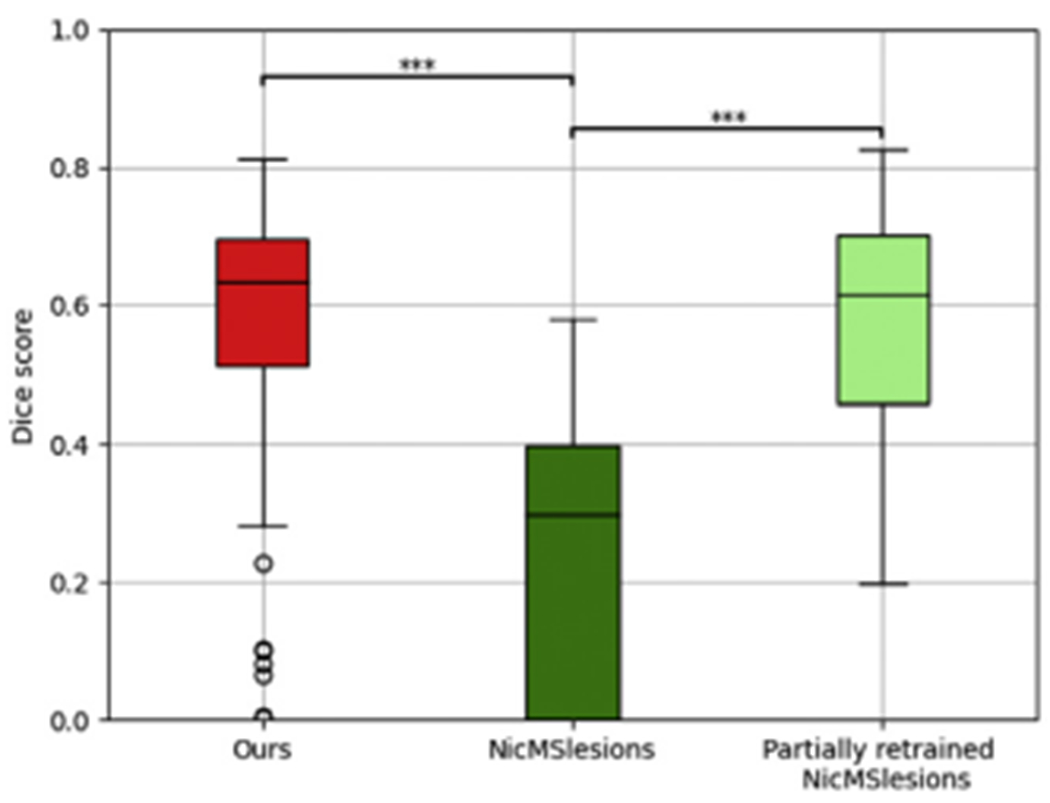
Lesion segmentation performance in terms of Dice overlap with manual raters on the ISBI dataset for the proposed method, NicMsLesions, and NicMsLesions with partial retraining (see text for details). Statistically significant differences between two methods, computed with a two-tailed paired *t*-test, are indicated by asterisks (“***” indicates *p*-value < 0.001).

In order to relate the performance of our method to the one obtained with the two benchmark methods, we also attempted to run LST-lga and NicMSLesions on this dataset. However, the preprocessing applied to the ISBI challenge data proved problematic for LST-lga, and we were not able to get any results with this method. Results for NicMSLesions in terms of Dice overlap are shown in Fig. 11, together with those obtained with the proposed method. It is clear that NicMSLesions suffers strongly from the domain shift between its training data and the ISBI data, a fact that was already reported in Valverde et al. (2019). For completeness, Fig. 11 also includes results for NicMSLesions when its network was updated on the ISBI training data as described in Valverde et al. (2019): different subsets of network parameters were retrained on the baseline scan of each of the five ISBI training subjects, and the combination that performed best on all 21 training images was retained. From the figure it can be seen that this partially retrained network has comparable performance to the proposed model, although the latter attains this performance without any retraining.

#### 5.3.3. Inter-rater variability

To evaluate the proposed method’s lesion segmentation performance in the context of human inter-rater variability, we took advantage of the availability of lesion segmentations by seven different raters in the MSSeg dataset. Table 3 shows the lesion segmentation performance in terms of average Dice overlap between each pair of the seven raters, and between each rater and the proposed method. On average, our method achieves a Dice overlap score of 0.57, which is slightly below the mean human raters’ range of [0.59, 0.69]. We note that this result is in line with those obtained in the MSSeg challenge (Commowick et al., 2018).

### 5.4. Whole-brain segmentation

Since no ground truth segmentations are available for a direct evaluation of the whole-brain segmentation component of our method, we performed an indirect validation, evaluating its potential for replacing lesion filling approaches that rely on manually annotated lesions, as well as its ability to replicate known atrophy patterns in MS. The results concentrate on the following 25 main neuroanatomical regions, segmented from T1w-FLAIR scans: left and right cerebral white matter, cerebellum white matter, cerebral cortex, cerebellum cortex, lateral ventricle, hippocampus, thalamus, putamen, pallidum, caudate, amygdala, nucleus accumbens and brain stem. To avoid cluttering, the quantitative results for left and right structures are averaged. We note that lesion segmentations are not merged into any of these brain structures (i.e., leaving “holes” in white matter), so that the results reflect performance only for the normal-appearing parts of structures.

#### 5.4.1. Comparison with lesion filling

It is well-known that white matter lesions can severely interfere with the quantification of normal-appearing structures when standard brain MRI segmentation techniques are used (Battaglini et al., 2012; Ceccarelli et al., 2012; Chard et al., 2010; Gelineau-Morel et al., 2012; Nakamura and Fisher, 2009; Vrenken et al., 2013). A common strategy is therefore to use a lesion-filling (Chard et al., 2010; Sdika and Pelletier, 2009) procedure, in which lesions are first manually segmented, their original voxel intensities are replaced with normal-appearing white matter intensities, and standard tools are then used to segment the resulting, preprocessed images. Using such a procedure with SAMSEG would yield whole-brain segmentations that can serve as “silver standard” benchmarks against which the results of the proposed method (which works directly on the original scans) can be compared. In practice, however, we have noticed that replacing lesion intensities, which is typically done in T1w only, did not work well in FLAIR in our experiments. Therefore, rather than explicitly replacing intensities, we obtained silver standard segmentations by simply masking out lesions during the SAMSEG processing, effectively ignoring lesion voxels during the model fitting.

We wished to interpret segmentation vs. silver standard discrepancies within the context of the human inter-rater variability associated with manually segmenting lesions. Therefore, we performed experiments on the MSSeg dataset, repeatedly re-computing the silver standard using each of the seven raters’ manual lesion annotations in turn. The results are shown in Tables 4 and 5 for Pearson correlation coefficients between estimated volumes and Dice segmentation overlap scores, respectively. Each line in these tables corresponds to one structure, showing the average consistency between the silver standard of each rater compared to that of the six other raters, as well as the average consistency between the proposed method’s segmentation and the silver standards of all raters. The results indicate that, in terms of Pearson correlation coefficient, the performance of our method falls within the range of inter-rater variability, albeit narrowly (average value 0.988 vs. inter-rater range [0.988, 0.992]). In terms of Dice scores, however, the method slightly underperforms compared to the inter-rater variability (average value 0.971 vs. inter-rater range [0.978, 0.980]).

#### 5.4.2. Detecting atrophy patterns in MS

**Fig. 12.**
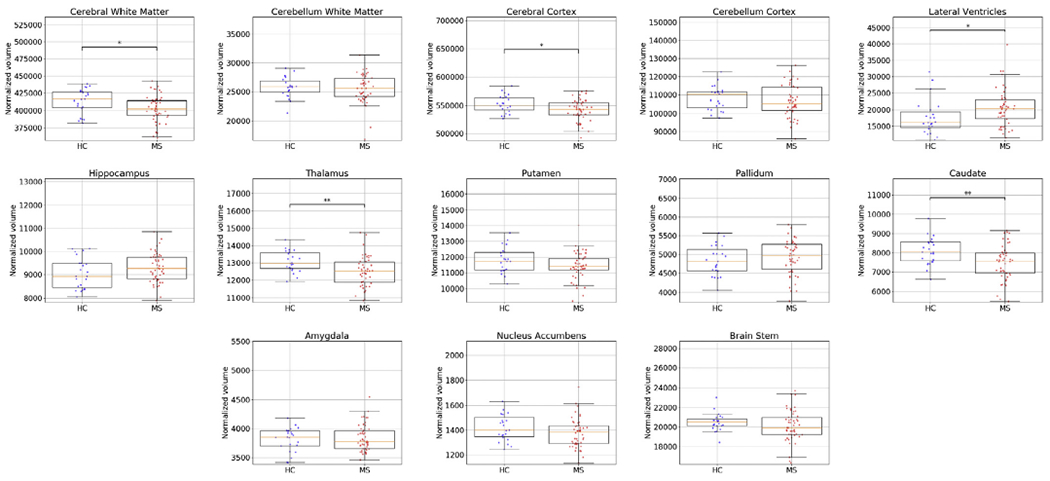
Differences between healthy controls (HC) and MS subjects in normalized volume estimates of various neuroanatomical structures, as detected by the proposed method on the Achieva dataset (23 HC subjects, 50 MS subjects, T1w-FLAIR input). Statistically significant differences between the two groups, computed with a Welch’s t-test, are indicated by asterisks (“**” for *p*-value < 0.01 and “*” for *p*-value < 0.05).

In a final analysis, we assessed whether previously reported volume reductions in specific brain structures in MS can automatically be detected with the proposed method. Towards this end, we segmented the 23 controls and the 50 MS subjects of the Achieva dataset, and compared the volumes of various structures between the two groups. Volumes were normalized for age, gender and total intracranial volume by regressing them out with a general linear model. The intracranial volume used for the normalization was computed by summing the volumes of all the structures, as segmented by the method, within the intracranial vault. The results are shown in Fig. 12. Although not all volumes showed significant difference between groups, well established differences were replicated. In particular, we demonstrated decreased volumes of cerebral white matter, cerebral cortex, thalamus and caudate (Azevedo et al., 2018; Chard et al., 2002; Houtchens et al., 2007) as well as an increased volume of the lateral ventricles (Zivadinov et al., 2016).

## 6. Discussion and conclusion

In this paper, we have proposed a method for the simultaneous segmentation of white matter lesions and normal-appearing neuroanatomical structures from multi-contrast brain MRI scans of MS patients. The method integrates a novel model for white matter lesions into a previously validated generative model for whole-brain segmentation. By using separate models for the shape of anatomical structures and their appearance in MRI, the algorithm is able to adapt to data acquired with different scanners and imaging protocols without needing to be retrained. We validated the method using four disparate datasets, showing robust performance in white matter lesion segmentation while simultaneously segmenting dozens of other brain structures. We further demonstrated that it can also be safely applied to MRI scans of healthy controls, and replicate previously documented atrophy patterns in deep gray matter structures in MS. The proposed algorithm is publicly available as part of the open-source neuroimaging package FreeSurfer.

By performing both whole-brain and white matter lesion segmentation at the same time, the method we propose aims to supplant the two-stage “lesion filling” procedure that is commonly used in morphometric studies in MS, in which lesions segmented in a first step are used to avoid biasing a subsequent analysis of normal-appearing structures with software tools developed for healthy brain scans. In order to evaluate whether our method is successful in this regard, we compared its whole-brain segmentation performance against the results obtained when lesions are segmented *a priori* by seven different human raters instead of automatically by the method itself. Our results show that the volumes of various neuroanatomical structures obtained when lesions are segmented automatically fall within the range of inter-rater variability, indicating that the proposed method may be used instead of lesion filling with manual lesion segmentations in large volumetric studies of brain atrophy in MS. When detailed spatial overlap is analyzed, however, we found that the automatic segmentation does not fully reach the performance obtained with human lesion annotation as measured by Dice overlap.

Like many other methods for MS lesion segmentation, the method proposed here produces a spatial map indicating in each voxel its probability of belonging to a lesion, which can then be thresholded to obtain a final lesion segmentation. Although in our experience good results can be obtained by using the same threshold value across datasets (e.g., *γ* = 0.5), changing this value allows one to adjust the trade-off between false positive and false negative lesion detections. Since some MRI sequences and scanners will depict lesions with a higher contrast than others, and because there is often considerable disagreement between human experts regarding the exact extent of lesions (Zijdenbos et al., 1998), in our implementation we therefore expose this threshold value as an optional, tunable parameter to the end-user. Suitable threshold values can be found by visually inspecting the lesion segmentations of a few cases or, in large-scale studies, using cross-validation as we did in our experiments.

By providing the ability to robustly and efficiently segment multicontrasts scans of MS patients across a wide range of imaging equipment and protocols, the software tool presented here may help facilitate large cohort studies aiming to elucidate the morphological and temporal dynamics underlying disease progression and accumulation of disability in MS. Furthermore, in current clinical practice, high-resolution multicontrast images, which can be used to increase the accuracy of lesion segmentation, represent a significantly increased burden for the neuroradiologist to read, and are hence frequently not acquired. The emergence of robust, multi-contrast segmentation tools such as ours may help break the link between the resolution and number of contrasts of the acquired data and the human time needed to evaluate it, thus potentially increasing the accuracy of the resulting measures.

The ability of the proposed method to automatically tailor its appearance models for specific datasets makes it very flexible, allowing it to seamlessly take advantage of novel, potentially more sensitive and specific MRI acquisitions as they are developed. Although not extensively tested, the proposed method should make it possible to, with minimal adjustments, segment data acquired with advanced research sequences such as MP2RAGE (Marques et al., 2010), DIR (Redpath and Smith, 1994), FLAIR^2^ (Wiggermann et al., 2016) or T2* (Anderson et al., 2001), both at conventional and at ultra-high magnetic field strengths. We are currently pursuing several extensions of the proposed method, including the ability to go on and create cortical surfaces and parcellations in FreeSurfer, as well as a dedicated version for longitudinal data (Cerri et al., 2020).

## Supplementary Material

1

## Figures and Tables

**Table 1:** Summary of the datasets used in our experiments.

Dataset	Scanner	Modality	Voxel resolution [mm]	Subjects
MSSeg	Philips Ingenia 3T	3D FLAIR	0.74×0.74×0.7	5
		3D T1w	0.74×0.74×0.85	
		3D T1c	0.74×0.74×0.85	
		2D T2w	0.45×0.45×3	
		2D PD	0.45×0.45×3	

	Siemens Aera 1.5T	3D FLAIR	1.03×1.03×1.25	5
		3D T1w	1.08×1.08×0.9	
		3D T1c	1.08×1.08×0.9	
		2D T2w	0.72×0.72×4 (Gap: 1.2)	
		2D PD	0.72×0.72×4 (Gap: 1.2)	

	Siemens Verio 3T	3D FLAIR	0.5×0.5×1.1	5
		3D T1w	1×1×1	
		3D T1c	1×1×1	
		2D T2w	0.69×0.69×3	
		2D PD	0.69×0.69×3	

Trio	Siemens Trio 3T	2D FLAIR	0.7×0.7×4	40
		3D T1w	1×1×1	
		2D T2w	0.7×0.7×4	

Achieve	Philips Achieva 3T	3D FLAIR	1×1×1	73
		3D T1w	0.85×0.85×0.85	
		3D T2w	0.85×0.85×0.85	

ISBI	Philips 3T	2D FLAIR	0.82×0.82×2.2	14
		3D T1w	0.82×0.82×1.17	
		2D T2w	0.82×0.82×2.2	
		2D PDw	0.82×0.82×2.2	

**Table 2:** Comparison in terms of lesion segmentation performance between the proposed method and a method where the lesion shape model was intentionally removed. Results are expressed in terms of mean ±standard deviation of Dice overlap, precision and recall for different ranges of lesion load. Lesion segmentations were computed across three different datasets (MSSeg, Trio and Achieva) on T1w-FLAIR input.

Lesion load	Dice		Precision		Recall	
	Shape model	No shape model	Shape model	No shape model	Shape model	No shape model
(0, 2] [ml]	0.42 (±0.10)	0.38 (±0.10)	0.32 ( ±0.12)	0.24 (±0.07)	0.28 (±0.09)	0.24 (±0.07)
(2, 10] [ml]	0.50 (±0.13)	0.47 (±0.13)	0.37 (±0.13)	0.33 (±0.11)	0.34 (±0.12)	0.32 (±0.12)
(10, −) [ml]	0.70 (±0.11)	0.70 (±0.11)	0.62 (±0.20)	0.62 (±0.20)	0.55 (±0.12)	0.55 (±0.13)

(0, −) [ml]	0.57 (±0.16)	0.55 (±0.17)	0.46 (±0.20)	0.43 (±0.20)	0.42 (±0.16)	0.40 (±0.16)

**Table 3:** Comparison of lesion segmentation performance in terms of average Dice score between each pair of the seven raters of the MSSeg dataset, and between each rater and the proposed method (T1w-FLAIR input).

	R1	R2	R3	R4	R5	R6	R7	Ours

R1	–	0.68	0.59	0.70	0.75	0.59	0.59	0.54
R2	0.68	–	0.59	0.71	0.72	0.60	0.57	0.56
R3	0.59	0.59	–	0.57	0.59	0.60	0.63	0.60
R4	0.70	0.71	0.57	–	0.90	0.57	0.54	0.53
R5	0.75	0.72	0.59	0.90	–	0.59	0.57	0.55
R6	0.59	0.60	0.60	0.57	0.59	–	0.61	0.57
R7	0.59	0.57	0.63	0.54	0.57	0.61	–	0.60

Avg	**0.65**	**0.64**	**0.60**	**0.66**	**0.69**	**0.60**	**0.59**	**0.57**

**Table 4:** Average Pearson correlation coefficients of brain structure volume estimates between the silver standard of each rater compared to that of the six other raters in the MSSeg dataset, as well as the average consistency between the proposed method's segmentation and the silver standards of all raters (T1w-FLAIR input). Each line shows an average across raters for a specific brain structure.

	R1	R2	R3	R4	R5	R6	R7	Ours

Cerebral White Matter	0.992	0.992	0.991	0.993	0.993	0.993	0.987	0.989
Cerebellum White Matter	0.994	0.997	0.997	0.996	0.997	0.997	0.997	0.989
Cerebral Cortex	0.997	0.999	0.999	0.999	0.999	0.999	0.999	0.997
Cerebellum Cortex	0.999	0.999	0.997	0.999	0.997	0.999	0.999	0.999
Lateral Ventricles	0.996	0.995	0.996	0.997	0.998	0.994	0.996	0.992
Hippocampus	0.982	0.989	0.987	0.979	0.977	0.979	0.984	0.981
Thalamus	0.998	0.997	0.998	0.998	0.998	0.997	0.997	0.996
Putamen	0.999	0.999	0.999	0.999	0.999	0.999	0.999	0.996
Pallidum	0.988	0.993	0.993	0.994	0.993	0.994	0.990	0.989
Caudate	0.994	0.993	0.987	0.990	0.995	0.989	0.993	0.985
Amygdala	0.953	0.967	0.970	0.973	0.941	0.957	0.972	0.963
Accumbens	0.985	0.987	0.987	0.966	0.989	0.953	0.988	0.971
Brain Stem	0.991	0.994	0.990	0.992	0.992	0.988	0.992	0.989

Average	**0.990**	**0.992**	**0.992**	**0.990**	**0.990**	**0.988**	**0.992**	**0.988**

**Table 5:** Same as Table 4, but with Dice segmentation overlap scores. Each line shows an average across raters – similar to the last row of Table 3 – for a specific brain structure.

	R1	R2	R3	R4	R5	R6	R7	Ours

Cerebral White Matter	0.982	0.982	0.982	0.983	0.983	0.982	0.981	0.978
Cerebellum White Matter	0.987	0.987	0.987	0.987	0.988	0.987	0.987	0.983
Cerebral Cortex	0.989	0.990	0.989	0.990	0.989	0.989	0.989	0.986
Cerebellum Cortex	0.996	0.996	0.995	0.996	0.996	0.995	0.995	0.994
Lateral Ventricles	0.972	0.970	0.972	0.974	0.976	0.971	0.971	0.954
Hippocampus	0.975	0.972	0.971	0.973	0.974	0.972	0.972	0.965
Thalamus	0.980	0.981	0.981	0.982	0.981	0.982	0.981	0.975
Putamen	0.987	0.987	0.988	0.988	0.988	0.988	0.987	0.980
Pallidum	0.985	0.985	0.986	0.986	0.986	0.986	0.985	0.978
Caudate	0.961	0.957	0.956	0.961	0.964	0.957	0.954	0.937
Amygdala	0.973	0.972	0.972	0.973	0.972	0.972	0.972	0.967
Accumbens	0.957	0.958	0.960	0.960	0.960	0.943	0.960	0.945
Brain Stem	0.987	0.986	0.984	0.986	0.986	0.986	0.986	0.983

Average	**0.979**	**0.979**	**0.979**	**0.980**	**0.980**	**0.978**	**0.978**	**0.971**

## Footnotes

1 Although selectively fusing several automatic methods has recently been shown to approach human performance (Carass et al., 2020).

2 http://freesurfer.net/.

3 http://www.xinapse.com/

4 https://www.applied-statistics.de/lst.html

5 https://www.fil.ion.ucl.ac.uk/spm/software/spm12

6 https://github.com/sergivalverde/nicMsLesions

7 https://smart-stats-tools.org/lesion-challenge

8 https://smart-stats-tools.org/lesion-challenge

## Appendix A. Parameter optimization in SAMSEG

We here describe how we perform the optimization of *p*(***θ***|**D**) with respect to ***θ*** and in the original SAMSEG model. We follow a coordinate ascent approach, in which a limited-memory BFGS optimization of ***θ*_l_** is interleaved with a generalized EM (GEM) optimization of the remaining parameters ***θ*_d_**. The GEM algorithm was derived in (Van Leemput et al., 1999) based on (Wells et al., 1996), and is repeated here for the sake of completeness. It iteratively constructs a tight lower bound to the objective function by computing the soft label assignments *ω*_*i,k*_ based on the current estimate of ***θ*_d_** (Eq. (2)), and subsequently improves the lower bound (and therefore the objective function) using the following set of analytical update equations for these parameters :

$$\mu_{k}\leftarrow m_{k}\;\text{and}\;\Sigma_{k}\leftarrow V_{k},\forall_{k}\;\left(\begin{array}{c} c_{1}\\ \vdots \\ c_{N} \end{array}\right)\leftarrow {\left(\begin{array}{ccc} A^{T}S_{1,1}A & \cdots & A^{T}S_{1,N}A\\ \vdots & \ddots & \vdots \\ A^{T}S_{N,1}A & \cdots & A^{T}S_{N,N}A \end{array}\right)}^{-1}\left(\begin{array}{c} A^{T}\left(\sum_{n=1}^{N}S_{1,n}r_{1,n}\right)\\ \vdots \\ A^{T}\left(\sum_{n=1}^{N}S_{N,n}r_{N,n}\right) \end{array}\right),$$

where

$$m_{k}=\frac{\sum_{i=1}^{I}\omega_{i,k}\left(d_{i}-C\phi_{i}\right)}{N_{k}}\;\mathrm{with}\;N_{k}=\sum_{i=1}^{I}\omega_{i,k},$$

$$V_{k}=\frac{\sum_{i=1}^{I}\omega_{i,k}\left(d_{i}-C\phi_{i}-m_{k}\right){\left(d_{i}-C\phi_{i}-m_{k}\right)}^{T}}{N_{k}},$$

$$A=\left(\begin{array}{ccc} \phi_{1}^{1} & \cdots & \phi_{p}^{1}\\ \vdots & \ddots & \vdots \\ \phi_{1}^{I} & \cdots & \phi_{P}^{I} \end{array}\right),\;S_{m,n}=\mathrm{diag}\left(s_{i}^{m,n}\right),\;r_{m,n}=\left(\begin{array}{c} r_{1}^{m,n}\\ \vdots \\ r_{I}^{m,n} \end{array}\right)$$

and

$$s_{i}^{m,n}=\sum_{k=1}^{K}s_{i,k}^{m,n},\;s_{i,k}^{m,n}=\omega_{i,k}{\left(\sum_{k}^{-1}\right)}_{m,n},\;r_{i}^{m,n}=d_{i}^{n}-\frac{\sum_{l=1}^{K}s_{i,k}^{m,n}{\left(\mu_{k}\right)}_{n}}{\sum_{l=1}^{K}s_{i,k}^{m,n}}.$$

## Appendix B. Parameter optimization

Here we describe how we perform the optimization of *p*(***θ, θ**_les_*|**D**) with respect to ***θ*** and ***θ**_les_* in the augmented model of Sec. 3 with the decoder outputs *f_i_*(**h**) all clamped to value 1. In that case, the model can be reformulated in the same form as the original SAMSEG model, so that the same optimization strategy can be used. In particular, lesions can be considered to form an extra class (with index *K* + 1) in a SAMSEG model with *K* + 1 labels, provided that the mesh vertex label probabilities

$${\tilde{\alpha }}_{j}^{k}=\{\begin{array}{cc} \beta_{j} & \mathrm{if}\;k=K+1\;\left(\mathrm{lesion}\right),\\ \alpha_{j}^{k}\left(\beta_{j}-1\right) & \mathrm{otherwise}. \end{array}$$

are used instead of the original $\alpha_{j}^{k}$’s in the atlas interpolation model of Eq. (1).

The optimization described in Appendix A does require one modification because of the prior *p*(***θ**_les_*|***θ*_d_**) binding the means and variances of the WM and lesion classes together. The following altered update equations for these parameters guarantee that the EM lower bound, and therefore the objective function, is improved in each iteration of the GEM algorithm:

$$\mu_{WM}\leftarrow {\left(N_{WM}I+\frac{vN_{WM}}{v+N_{WM}}\Sigma_{WM}\Sigma_{les}^{-1}\right)}^{-1}\;(N_{WM}m_{WM}+\frac{vN_{WM}}{v+N_{WM}}\Sigma_{WM}\Sigma_{les}^{-1}m_{les}),$$

$$\Sigma_{WM}\leftarrow \frac{N_{WM}V_{WM}+\Sigma_{les}\Sigma_{WM}^{-1}\Psi_{les}}{N_{WM}+N_{les}+N+2},$$

$$\mu_{les}\leftarrow \frac{N_{les}m_{les}+v\mu_{WM}}{N_{les}+v},$$

$$\Sigma_{les}\leftarrow \frac{\Psi_{les}+v\kappa \Sigma_{WM}}{N_{les}+v},$$

where $\Psi_{les}=\frac{N_{les}v}{N_{les}+v}\left(m_{les}-\mu_{WM}\right){\left(m_{les}-\mu_{WM}\right)}^{T}+N_{les}V_{les}.$

## Appendix C. Estimating lesion probabilities

We here describe how we we approximate $p\left(z_{i}=1|d_{i},\hat{\theta }\right)$ using Monte Carlo sampling. We use a Markov chain Monte Carlo (MCMC) approach to sample triplets {$\theta_{les}^{\left(s\right)},z^{\left(s\right)},h^{\left(s\right)}$} from the distribution $p(\theta_{les},z,h|D,\hat{\theta }):$ Starting from an initial lesion segmentation **z**^(0)^ obtained from the parameter estimation procedure described in Appendix B, we use a blocked Gibbs sampler in which each variable is updated conditioned on the other ones:

$$\Sigma_{les}^{\left(s+1\right)}∼p(\Sigma_{les}|D,\hat{\theta },z^{\left(s\right)})=\;\mathrm{IW}(\Sigma_{les}|\psi_{les}^{\left(s\right)}+v\kappa {\hat{\Sigma }}_{W,M},N_{les}^{\left(s\right)}+v-N-2)$$

$$\mu_{les}^{\left(s+1\right)}∼p\left(\mu_{les}|D,\hat{\theta },z^{\left(s\right)},\Sigma_{les}^{\left(s+1\right)}\right)=\;N\left(\mu_{les}|\frac{N_{les}^{\left(s\right)}m_{les}^{\left(s\right)}+v{\hat{\mu }}_{WM}}{N_{les}^{\left(s\right)}+v},\frac{\Sigma_{les}^{\left(s+1\right)}}{N_{les}^{\left(s\right)}+v}\right)$$

$$h^{\left(s+1\right)}∼p\left(h|z^{\left(s\right)}\right)≃N\left(h|\mu_{\upsilon }\left(z^{\left(s\right)}\right),\mathrm{diag}\left(\sigma_{\upsilon }^{2}\left(z^{\left(s\right)}\right)\right)\right)$$

$$z^{\left(s+1\right)}\sim p\left(z|D,\hat{\theta },h^{\left(s+1\right)},\theta_{les}^{\left(s+1\right)}\right)=\prod_{i=1}^{I}p\left(z_{i}|d_{i},\hat{\theta },h^{\left(s+1\right)},\theta_{les}^{\left(s+1\right)}\right),$$

where we use the encoder variational approximation obtained during the training of the lesion shape model (see Sec. 3.1.2) to sample from **h** in the next-to-last step, and

$$p\left(z_{i}=1{|d}_{i},\hat{\theta },h,\theta_{les}\right)=\frac{N\left(d_{i}{|\mu }_{les}+C\phi_{i},\Sigma_{les}\right)f_{i}\left(h\right)\rho_{i}\left({\hat{\theta }}_{l}\right)}{\sum_{l_{i}=1}^{K}\sum_{z_{i}^{\prime }=0}^{1}p\left(d_{i}|l_{i},z_{i}^{\prime },{\hat{\theta }}_{l},\theta_{les}\right)p\left(z_{i}^{\prime }|{\hat{\theta }}_{l},h\right)p\left(l_{i}|{\hat{\theta }}_{l}\right)}$$

in the last step. In these equations, the variables $N_{les}^{\left(s\right)}$, $m_{les}^{\left(s\right)}$, $V_{les}^{\left(s\right)}$ and $\Psi_{les}^{\left(s\right)}$ are as defined before, but using voxel assignments $w_{i,\mathrm{les}}=z_{i}^{\left(s\right)}$. Once *S* samples are obtained, we approximate $p\left(z_{i}=1|d_{i},\hat{\theta }\right)$ as

$$p\left(z_{i}=1|d_{i},\hat{\theta }\right)≃\frac{1}{S}\sum_{s=1}^{S}p\left(z_{i}=1{|d}_{i},\hat{\theta },h^{\left(s\right)},\theta_{les}^{\left(s\right)}\right).$$

In our implementation, we use *S* = 50 samples, obtained after discarding the first 50 sweeps of the sampler (so-called “burn-in” phase). The algorithm repeatedly invokes the decoder and encoder networks of the lesion shape model described in Sec. 3.1.2. Since this shape model was trained in a specific isotropic space, the algorithm requires transitioning between this training space and subject space using an affine transformation. This is accomplished by resampling the input and output of the encoder and decoder, respectively, using linear interpolation.
