# Supplementary material for: A contrast-adaptive method for simultaneous whole-brain and lesion segmentation in multiple sclerosis
Source: Neuroimage. Author manuscript; Available in PMC 2021 Feb 3. (PMC7856304; doi:10.1016/j.neuroimage.2020.117471)
Supplement: 1 [file NIHMS1659126-supplement-1.pdf]

Supplementary Material for
“A Contrast-Adaptive Method for Simultaneous Whole-Brain and Lesion Segmentation in Multiple Sclerosis ”

September 4, 2020

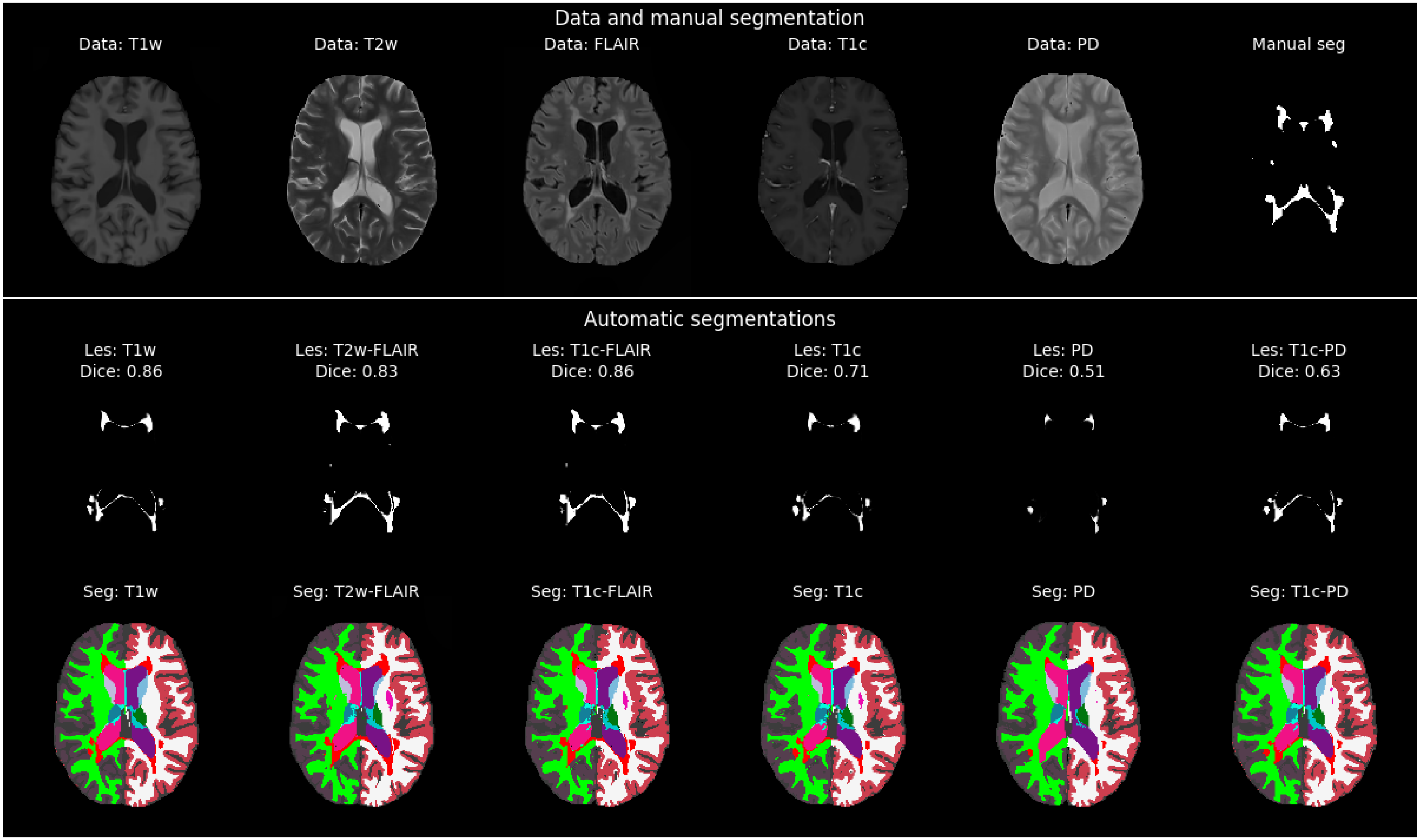

Fig. 1: Contrast-adaptiveness of the proposed method to different combinations of input modalities. Segmentations are shown for one subject of the MSSeg dataset. The top row shows slices of the data and the manual lesion annotation; the middle row shows the lesion probability map and Dice score computed by the proposed method for specific input combinations; and the bottom row shows the corresponding complete segmentations produced by the method.

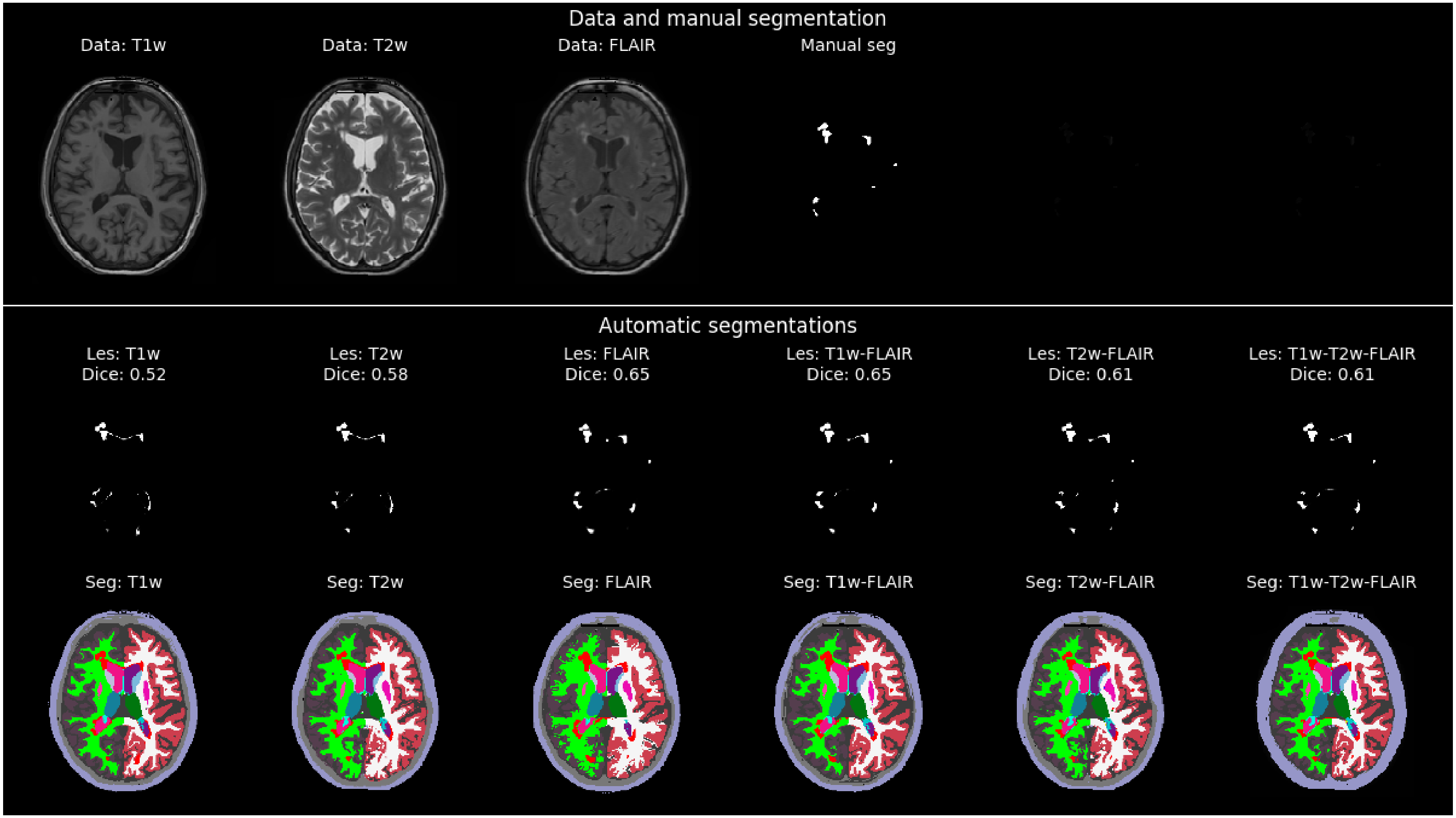

Fig. 2: Contrast-adaptiveness of the proposed method to different combinations of input modalities. Segmentations are shown for one subject of the Trio dataset. The top row shows slices of the data and the manual lesion annotation; the middle row shows the lesion probability map and Dice score computed by the proposed method for specific input combinations; and the bottom row shows the corresponding complete segmentations produced by the method.

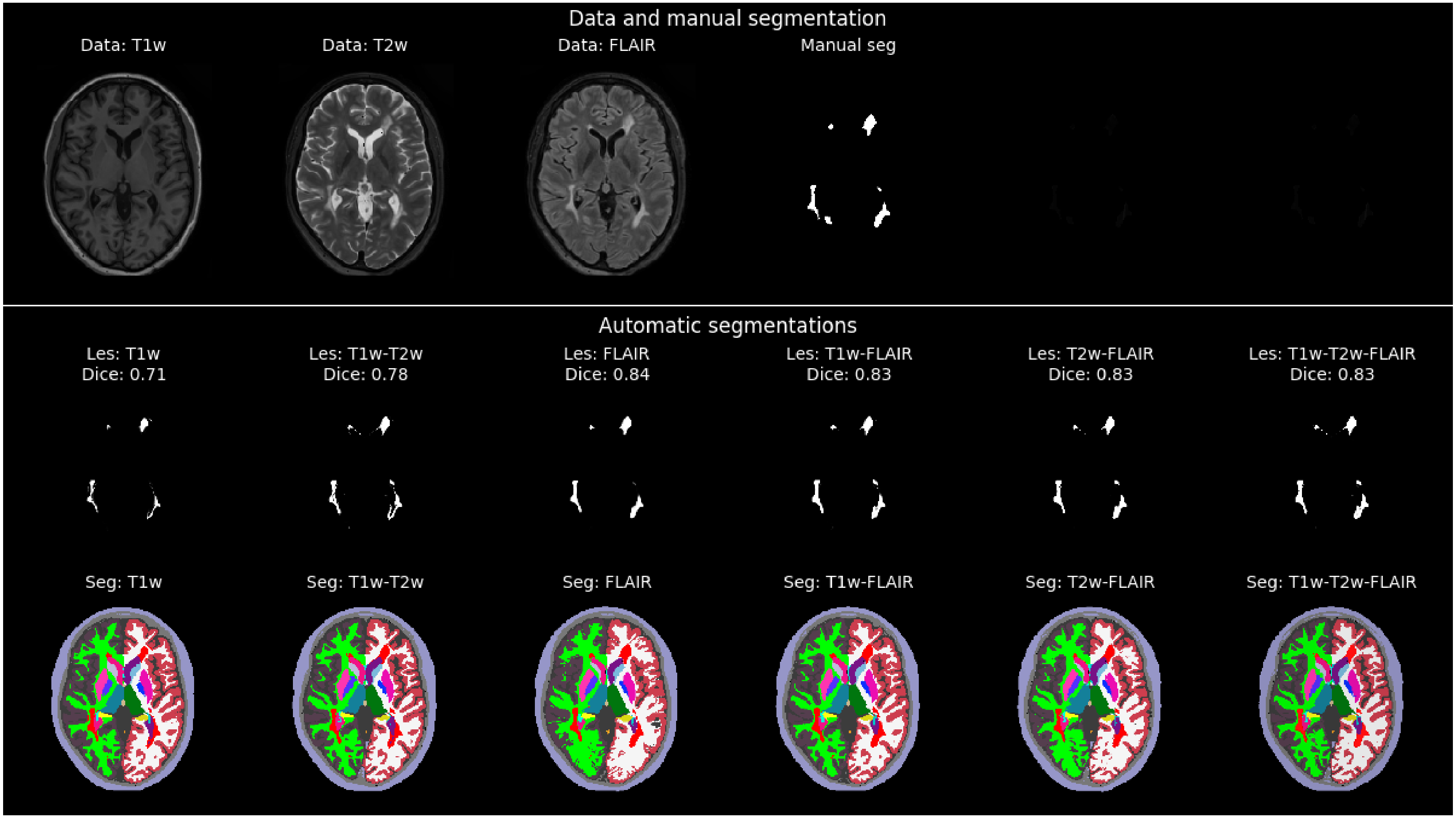

Fig. 3: Contrast-adaptiveness of the proposed method to different combinations of input modalities. Segmentations are shown for one subject of the Achieva dataset. The top row shows slices of the data and the manual lesion annotation; the middle row shows the lesion probability map and Dice score computed by the proposed method for specific input combinations; and the bottom row shows the corresponding complete segmentations produced by the method.

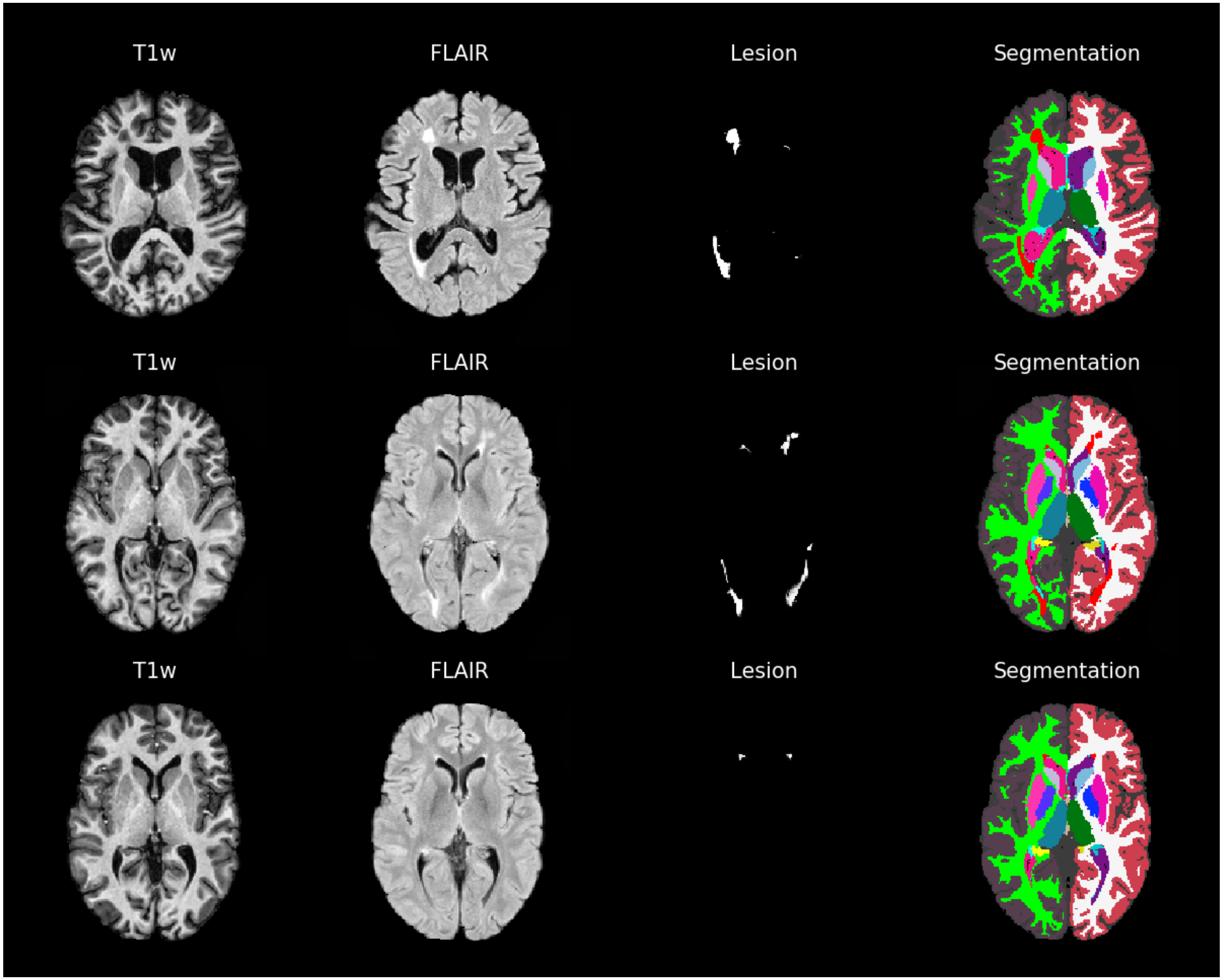

Fig. 4: Segmentations of three subjects of the ISBI dataset from the proposed method on T1w-FLAIR input. From top to bottom: high, median and low score lesion performance obtained on the website evaluation platform of the ISBI challenge. From left to right: T1w, FLAIR, lesion probability map, whole-brain segmentation.
